# A statistical analysis of the novel coronavirus (COVID-19) in Italy and Spain

**DOI:** 10.1371/journal.pone.0249037

**Published:** 2021-03-25

**Authors:** Jeffrey Chu

**Affiliations:** School of Statistics, Renmin University of China, Beijing, China; Faculty of Science, Ain Shams University (ASU), EGYPT

## Abstract

The novel coronavirus (COVID-19) that was first reported at the end of 2019 has impacted almost every aspect of life as we know it. This paper focuses on the incidence of the disease in Italy and Spain—two of the first and most affected European countries. Using two simple mathematical epidemiological models—the Susceptible-Infectious-Recovered model and the log-linear regression model, we model the daily and cumulative incidence of COVID-19 in the two countries during the early stage of the outbreak, and compute estimates for basic measures of the infectiousness of the disease including the basic reproduction number, growth rate, and doubling time. Estimates of the basic reproduction number were found to be larger than 1 in both countries, with values being between 2 and 3 for Italy, and 2.5 and 4 for Spain. Estimates were also computed for the more dynamic effective reproduction number, which showed that since the first cases were confirmed in the respective countries the severity has generally been decreasing. The predictive ability of the log-linear regression model was found to give a better fit and simple estimates of the daily incidence for both countries were computed.

## Introduction

The novel coronavirus (COVID-19) was widely reported to have first been detected in Wuhan (Hebei province, China) in December 2019. After the initial outbreak, COVID-19 continued to spread to all provinces in China and very quickly spread to other countries within and outside of Asia. At present, over 45 million cases of infected individuals have been confirmed in over 180 countries with in excess of 1 million deaths [1]. Although the foundations of this disease are very similar to the severe acute respiratory syndrome (SARS) virus that took hold of Asia in 2003, it is shown to spread much more easily and there currently exists no vaccine.

Since the first confirmed cases were reported in China, much of the literature has focused on the outbreak in China including the transmission of the disease, the risk factors of infection, and the biological properties of the virus—see for example key literature such as [2–6]. However, more recent literature has started to cover an increasing number of regions outside of China.

For example, studies covering the wider Asia region include: investigations into the outbreak on board the Diamond Princess cruise ship in Japan, using a Bayesian framework with a Hamiltonian Monte Carlo algorithm [7]; estimation of the ascertainment rate in Japan using a Poisson process [8]; modelling the evolution of the basic and effective reproduction numbers in South Korea using Susceptible-Infected-Susceptible models [9] and generalised growth models with varying growth rates [10]; modelling the basic reproduction number in India with a classical Susceptible-Exposed-Infectious-Recovered-type compartmental model [11]; forecasting numbers of cases in Indian states using deep learning-based models [12].

Analyses on North and South America have also used similar classical methods, for example [13] model the progression of the outbreak in the United States until the end of 2021 with the simple Susceptible-Infected-Recovered model, and [14] predict epidemic trends in Brazil and Peru using a logistic growth model and machine learning techniques. However, other studies include: analysis of the spatial variability of the incidence in the United States using spatial lag and error models, and geographically weighted regression [15]; estimation of the number of deaths in the United States using a modified logistic fault-dependent detection model [16]; estimating prevalence and infection rates across different states in the United States using a sample selection model [17]; investigating the relationship between social media communication and the incidence in Colombia using non-linear regression models.

Focusing on Africa, [18] simulate and predict the spread of the disease in South Africa, Egypt, Algeria, Nigeria, Senegal, and Kenya, using a modified Susceptible-Exposed-Infectious-Recovered model; [19] apply a six-compartmental model to model the transmission in South Africa; [20] predict the spread of the disease in West Africa using a deterministic Susceptible-Exposed-Infectious-Recovered model; [21] implement Autoregressive Integrated Moving Average models to forecast the prevalence of COVID-19 in East Africa; [22] predict the spread of the disease using travel history and personal contact in Nigeria through ordinary least squares regression; [23] use logistic growth and Susceptible-Infected-Recovered models to generate real-time forecasts of daily confirmed cases in Saudi Arabia.

Aside from many of the classical models mentioned above, recent developments in the econometrics and statistics literature have led to a number of new models that could potentially be applied in the modelling of infectious diseases. These include (but are not limited to) mixed frequency analysis, model selection and combination, and dynamic time warping. Mixed frequency analysis is an iterative approach proposed for dealing with the joint dynamics of time series data which are sampled at different frequencies [24]. In the economic literature, the common example is quarterly gross domestic product (GDP) and monthly inflation. [25] notes that studying the co-movements between mixed frequency data usually involves analysing the joint process sampled at a common low frequency, however, this can mis-specify the relationship. [24, 25] propose vector autoregressive models for mixed frequency analysis that operate at the highest sampling frequency of all the time series in the model. These models allow for the modelling of the joint dynamics of the dependent and independent variables using time disaggregation, where the low frequency variables are interpolated and time-aggregated into a higher frequency. In the context of infectious diseases, such models could be beneficial for modelling the relationship between higher frequency data such as the number of daily cases or deaths and lower frequency data relating to, say, weekly cases or deaths, news and information about health prevention measures, etc. [26, 27] propose the use of Bayesian Predictive Synthesis (BPS) for model selection and combination. They note that there are many scenarios that generate multiple, interrelated time series, where the dependence has a significant impact on decisions, policies, and their outcomes. In addition, methods need to learn and integrate information about forecasters and models, bias, etc. and how they change over time, to improve their accuracy [26]. Decision and policy makers often use multiple sources, models, and forecasters to generate forecasts, in particular, probabilistic density forecasts. However, although complex estimation methods may have useful properties for policy makers, large standard deviations may be a result of the complexity of the data, model, etc., and it may be difficult to know the source. The aim is to use the dependencies between time series to improve forecasts over multiple horizons for policy decisions [27]. For example, in the economic literature, setting interest rates based on utility or loss that account for inflation, real economy measures, employment, etc. BPS relates to a decision maker that accounts for multiple models as providers of “forecast data” to be used for prior-posterior updating. The decision maker learns over time about relationships between agents, forecasts, and dependencies, which are incorporated into the model, and dynamically calibrate, learn, and update weights for ranges of forecasts from dynamic models, with multiple lags and predictors [26]. In epidemiology, BPS could potentially be used in a similar context to analyse the dependency between various interrelated time series such as daily cases and deaths, hospital capacity, number vaccinations, etc. Different models and sources of data could then be combined and characterised in one single model improving the accuracy of forecasts. Dynamic time warping as noted by [28, 29] is a technique that has not been widely used outside of speech and gesture recognition. It can be used to identify the relation structure between two time series by describing their non-linear alignment with warping paths [28]. The procedure involves a local cost measure characterising the sum of the differences between pairs of realisations of data at each time point, where an optimal warping path gives the lowest total cost. The optimal path is found under a variable lead-lag structure, where the most suitable lag can then be found [28]. This then reveals and identifies the lead-lag effects between the time series data. Indeed, dynamic time warping has recently been used in the modelling of COVID-19 by [30]. [30] use the method to determine the lead-lag relation between the cumulative number of daily cases of COVID-19 in various countries, in addition to forecasting the future incidence in selected countries. This allows for the classification of countries as being in the early, middle, and late stages of an outbreak.

Controlling an infectious disease such as COVID-19 is an important, time-critical but difficult issue. The health of the global population is, perhaps, the most important factor as research is directed towards vaccines and governments scramble to implement public health measures to reduce the spread of the disease. In most countries around the world, these measures have come in the form of local or national lockdowns where individuals are advised or required to remain at home unless they have good reason not to—e.g. for educational or medical purposes, or if they are unable to work from home. However, the implications of trying to control COVID-19 are being felt not only by the health sector, but also in areas such as the economy, environment, and society.

As the number of cases of infected individuals has risen rapidly, there has been an increase in pressure on medical services as healthcare providers seek to test and diagnose infected individuals, in addition to the normal load of medical services that are offered in general. In many cases, trying to control COVID-19 has led to a backlog for and deprivation of other medical procedures [31], with healthcare providers needing to find a balance between the two. [32] note that this conflict may change the nature of healthcare with public and private health sectors working together more often. The implementation of restrictions on the movement of individuals has also led to many suggesting that anxiety and distress may lead to increased psychiatric disorders. These may be related to suicidal behaviour and morbidity and may have a long-term negative impact on the mental health of individuals [33, 34].

In addition to restrictions on the movement of individuals, governments have required most non-essential businesses to close. This has negatively impacted national economies with many businesses permanently closing leading to a significant increase in unemployment. Limits on travel have severely affected the tourism and travel industries, and countries and economies that are dependent on these for income. Whilst many of the implications of controlling COVID-19 on the economy are negative, there have been some positive changes as businesses adapt to the ‘new normal’. For example, the banking industry is dealing with increased credit risks, while the insurance industry is developing more digital products and pandemic-focused solutions [32]. The automotive industry is expected to see profits reduced by approximately $100 billion, which may be offset by the development of software subscription services of modern vehicles [32]. Some traditional office-based businesses have been able to reduce costs by shifting to remote working, while the restaurant industry has shifted towards takeaway and delivery services [32].

In terms of the environment, the limitations on businesses that have been able to continue operating throughout the epidemic has led to possible improvements in the environment—mainly from the reduction in pollution [35]. However, societal issues have been exacerbated. [32] note that the reduction in the labour force that has resulted from controlling for COVID-19 has affected ethnic minorities and women most significantly. Furthermore, in many countries health services employ more women than men creating a dilemma for working mothers—either leave the labour force and provide childcare for their families or remain in employment and pay extra costs for childcare.

In Europe, Italy and Spain were two of the first European countries to be significantly affected by COVID-19. However, the majority of the literature covering the two countries focuses on the clinical aspects of the disease, [36–40], with only a limited number exploring the prevalence of the disease, [41–43].

As as a result of this on going pandemic, new results and reports are being produced and published daily. Thus, our motivation stems from wanting to contribute to the statistical analysis of the incidence of COVID-19 in Italy and Spain, where the literature is limited. The main contributions of this paper are: i) to model the incidence of COVID-19 in Italy and Spain using simple mathematical models in epidemiology; ii) to provide estimates of basic measures of the infectiousness and severity of COVID-19 in Italy and Spain; iii) to investigate the predictive ability of simple mathematical models and provide simple forecasts for the future incidence of COVID-19 in Italy and Spain.

The contents of this paper are organised as follows. In the data section, we describe the incidence data used in the main analysis and provide a brief summary analysis. The method section outlines the Susceptible-Infectious-Recovered model and the log-linear model used to model the incidence of COVID-19, and introduces the basic reproduction number and effective reproduction number as measures of the infectiousness of diseases. In the results section, we present the main results for the fitted models and estimates of the measures of infectiousness, in addition to simple predictions for the future incidence of COVID-19. Some concluding remarks are given in the conclusion.

## Data

The data used in this analysis consists of the daily and cumulative incidence (confirmed cases) of COVID-19 for Italy and Spain (nationally), and their respective regions or autonomous provinces. For Italy, this data covers 21 regions for 37 days from 21st February 2020 to 28th March 2020, inclusive; for Spain, this data covers 19 regions for 34 days from 27th February to 31st March 2020, inclusive. The data for Italy was obtained from [44] where the raw data was sourced from the Italian Department of Civil Protection; the data for Spain was obtained from [45] where the raw data was sourced from the Spanish Ministry of Health. The starting dates for both sets of data indicate the dates on which the first cases were confirmed in each country, however, it should be noted that in some regions cases were not confirmed until after these dates. These particular time periods were chosen as they cover over one month since the initial outbreaks in both countries and were the most up to date data available at the time of writing. In the remainder of this section, we provide a simple exploratory analysis of the incidence data.

### Italy

**Fig 1:**
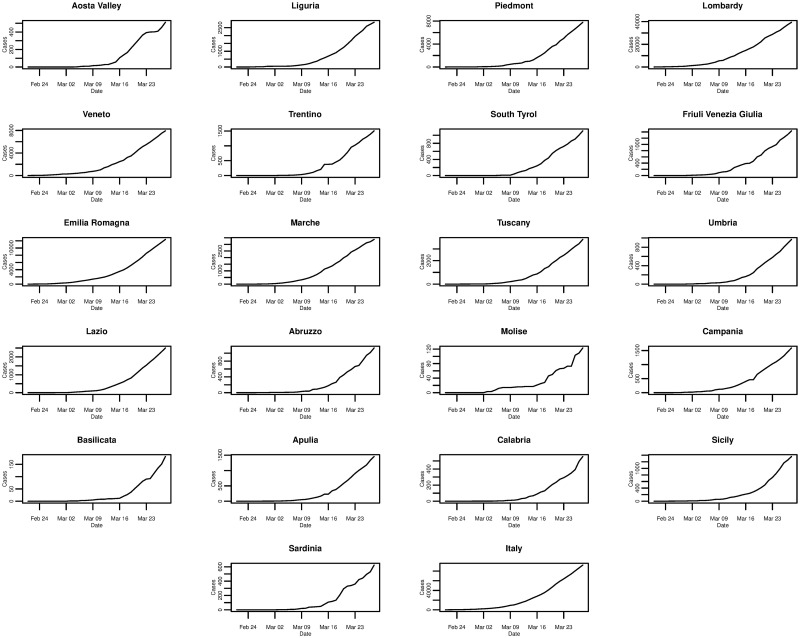
Daily cumulative incidence of the 21 Italian regions and Italy for the period of 21/02/2020 to 28/03/2020, inclusive.

**Fig 2:**
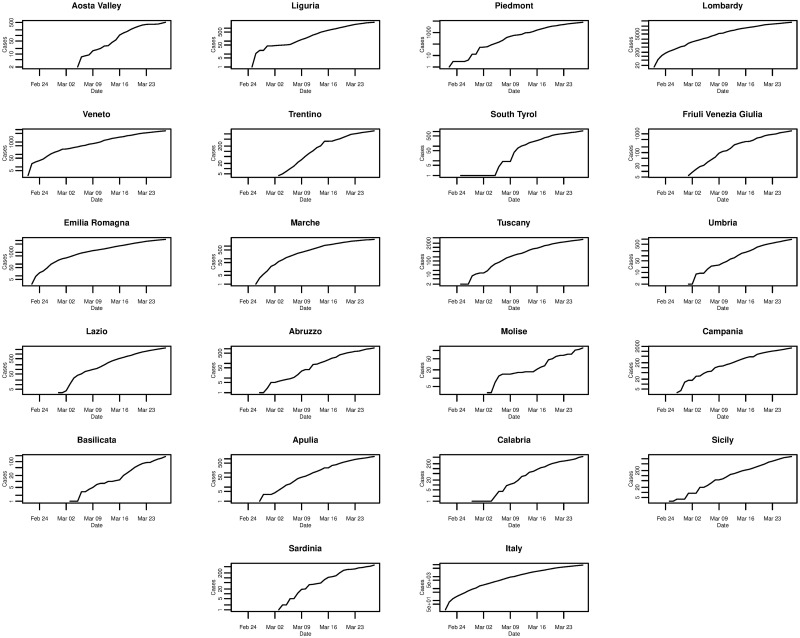
Daily cumulative incidence (log scale) of the 21 Italian regions and Italy for the period of 21/02/2020 to 28/03/2020, inclusive.

Fig 1 plots the daily cumulative incidence for Italy and its 21 regions over the whole sample period. All cumulative incidence appears to show an exponential trend, increasing slowly for the first 14 days after the first cases are confirmed before growing rapidly. Checking the same plot on a log-linear scale, shown in Fig 2, we find that the logarithm of cumulative incidence in some regions exhibits an approximate linear trend suggesting that cumulative incidence is growing exponentially. However, in the majority of regions (and nationally) this trend is not exactly linear, suggesting a slightly sub-exponential growth in cumulative incidence.

**Fig 3:**
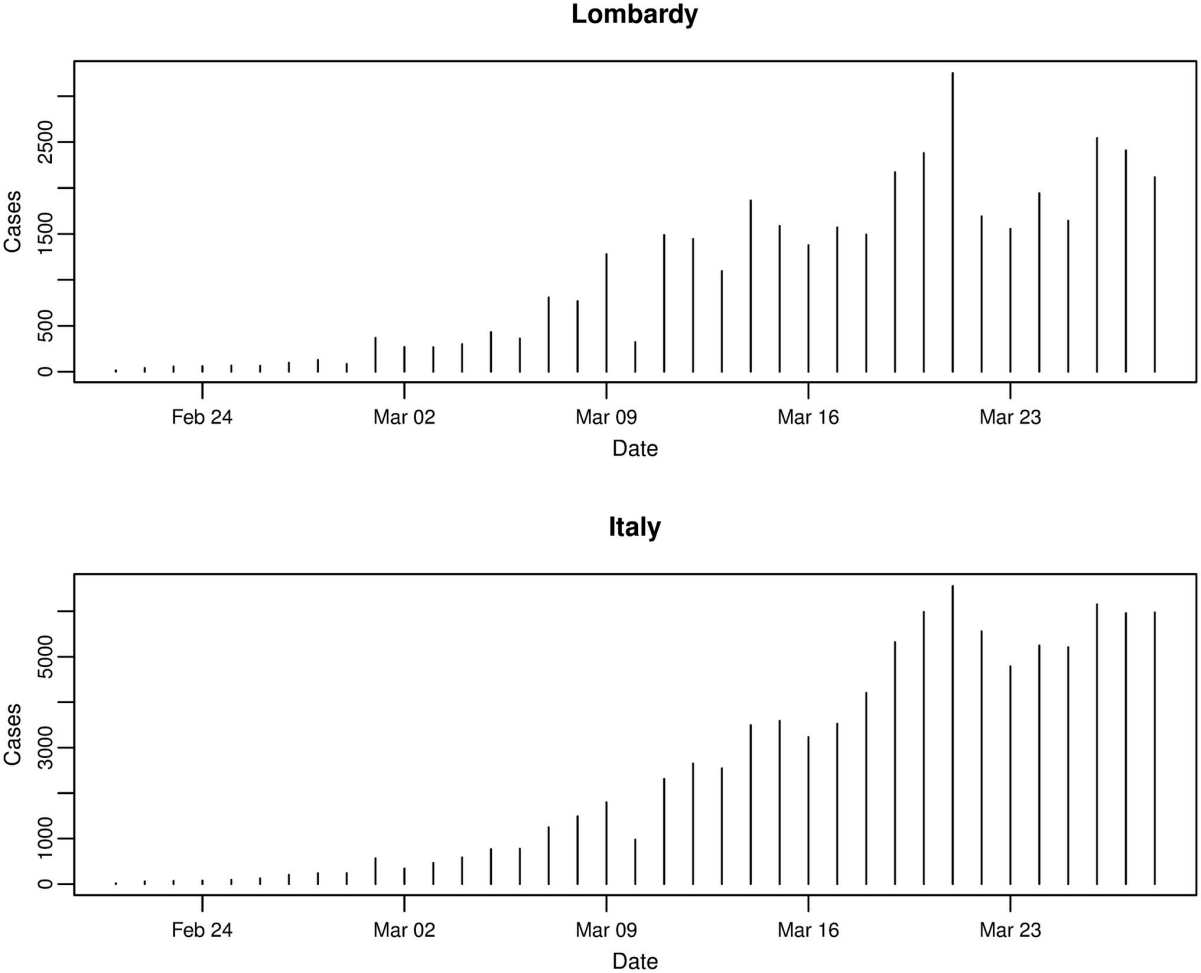
Daily incremental incidence of the Lombardy region and Italy for the period of 21/02/2020 to 28/03/2020, inclusive.

**Fig 4:**
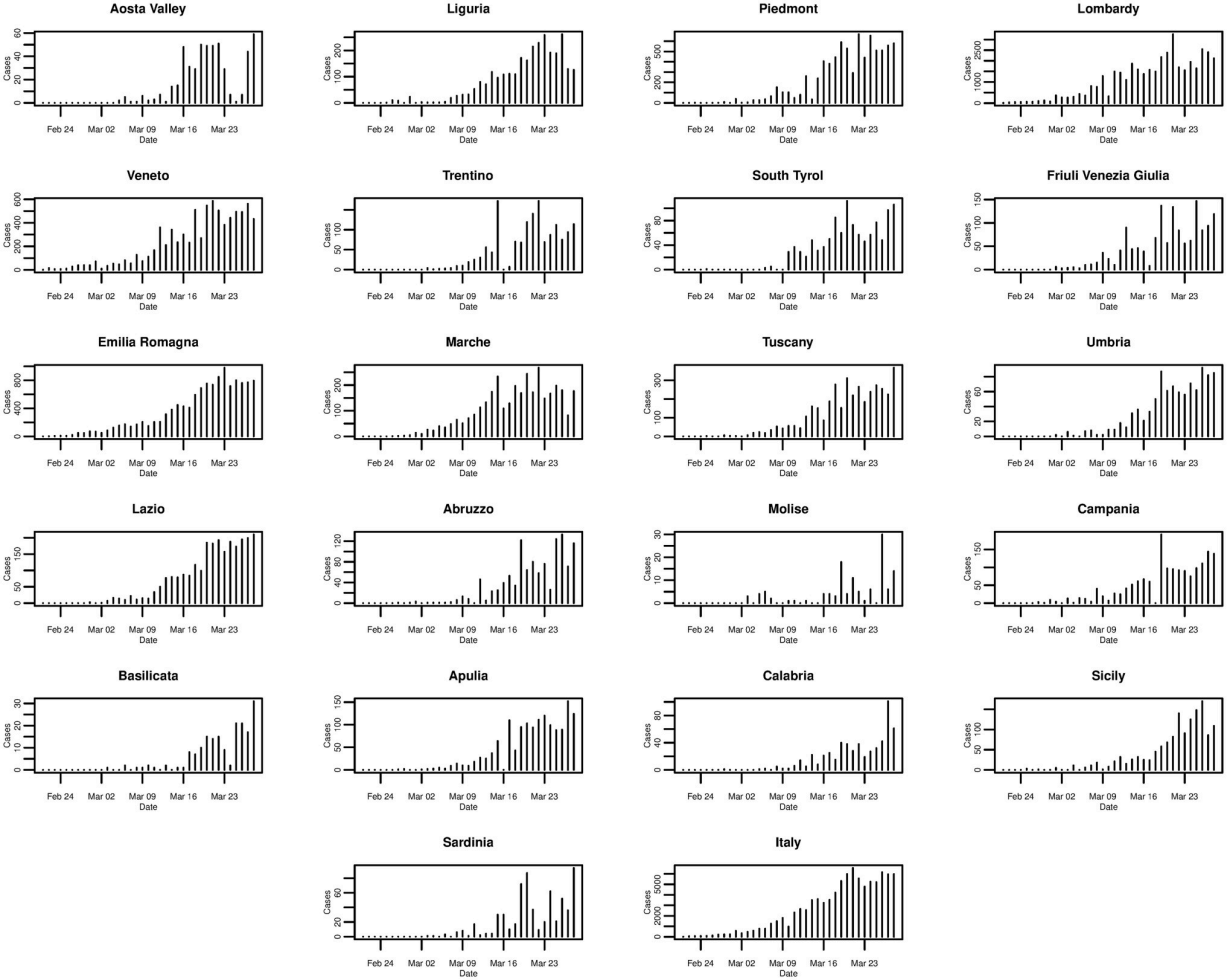
Daily incremental incidence of the 21 Italian regions and Italy for the period of 21/02/2020 to 28/03/2020, inclusive.

Of all the regions in Italy, the northern region of Lombardy is one of the worst affected and Fig 3 plots the daily incremental incidence for both Lombardy and Italy, respectively. In terms of the number of new cases confirmed each day, the trends are very similar and, again, possibly exponential until peaking around 21st March 2020 before levelling off. Comparing the trends for the other regions in Fig 4, it can be seen that other significantly affected northern regions such as Piedmont and Emilia-Romagna exhibit similarities to Lombardy—growing, peaking, and levelling around the same times. However, many other regions show some slight differences such as peaking at earlier or later dates, and even exhibiting an erratic trend.

**Fig 5:**
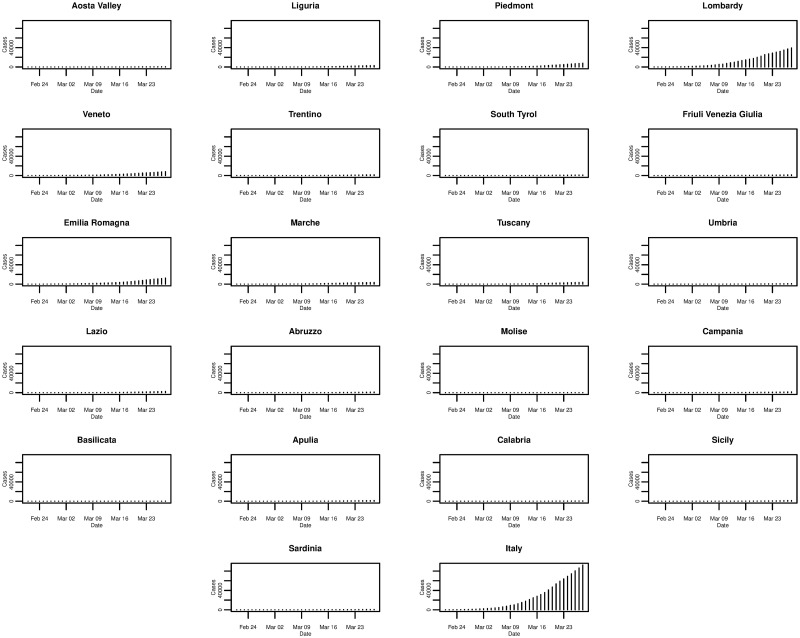
Daily incremental incidence (common scale) of the 21 Italian regions and Italy for the period of 21/02/2020 to 28/03/2020, inclusive.

In Fig 5, things are put in perspective when the cumulative incidence of all Italian regions are plotted on the same scale. It is clear that Lombardy is the most affected region contributing to the largest share of national cumulative incidence, and indeed it is the epicentre of the outbreak in Italy.

### Spain

**Fig 6:**
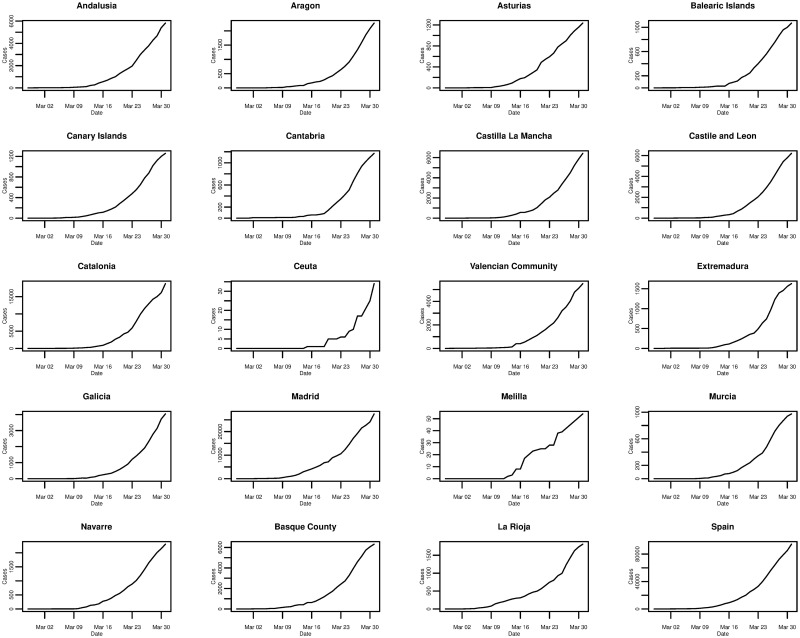
Daily cumulative incidence of the 19 Spanish regions and Spain for the period of 27/02/2020 to 31/03/2020, inclusive.

**Fig 7:**
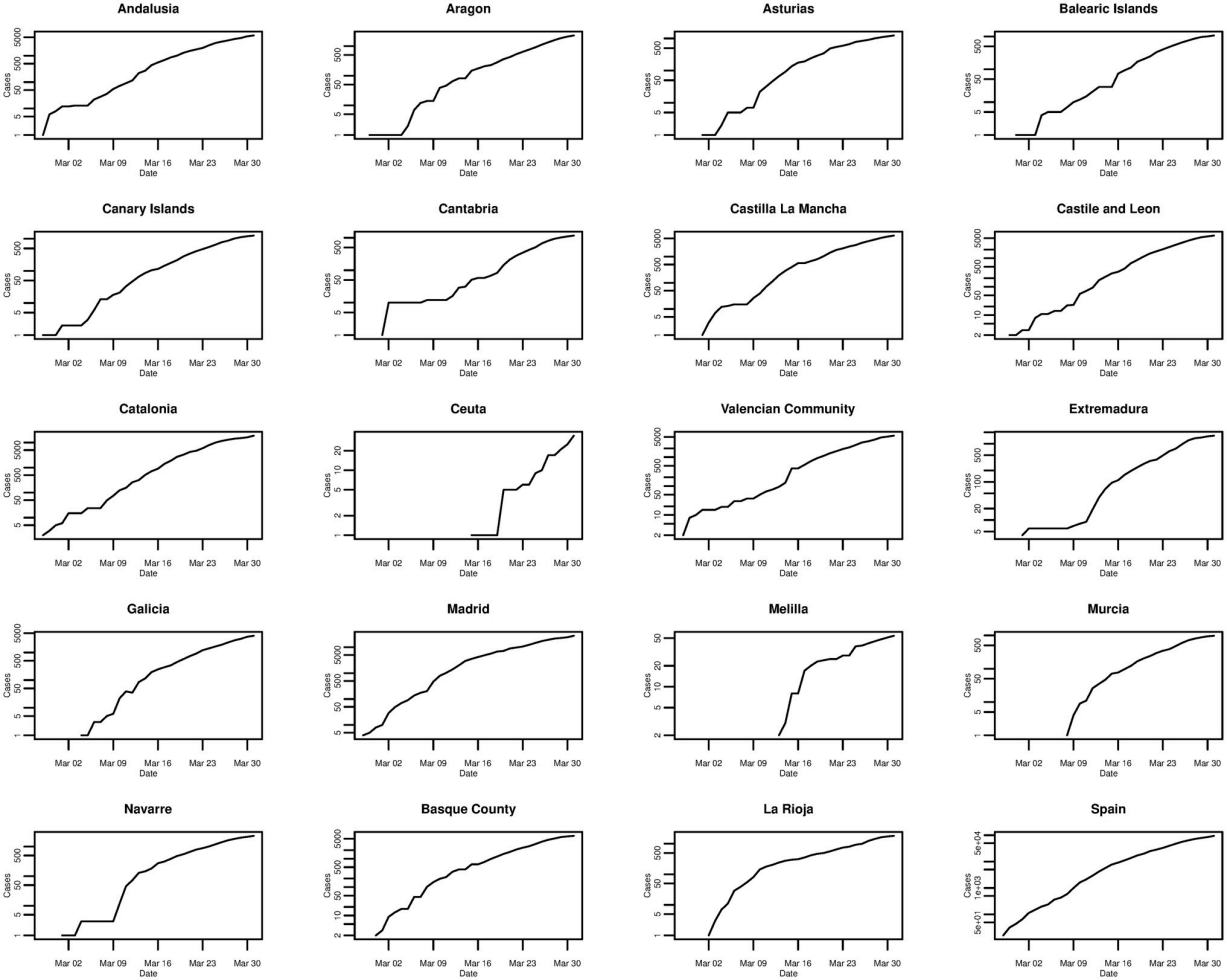
Daily cumulative incidence (log scale) of the 19 Spanish regions and Spain for the period of 27/02/2020 to 31/03/2020, inclusive.

In the case of Spain, Fig 6 plots the daily cumulative incidence nationally and for all 19 Spanish regions over the whole sample period. The trend appears to be exponential and is similar between regions, but is also similar to that of the daily cumulative incidence in Italy. On a log-linear scale, in Fig 7, the growth of the daily cumulative incidence appears to be closer to an exponential trend compared with Italy, due to the plots arguably exhibiting a more linear trend. It can be seen that there is a slight difference with Italy in that it appears as though most Spanish regions were affected at approximately the same time—when the country’s first cases were confirmed. This is reflected by the majority of plots starting from the very left of the x-axis, with the exception of the plots for a few regions such as Ceuta and Melilla. In Italy only a small number of regions were affected when the country’s first cases were confirmed, with the growth in cumulative incidence for the majority of the other regions coming later on.

**Fig 8:**
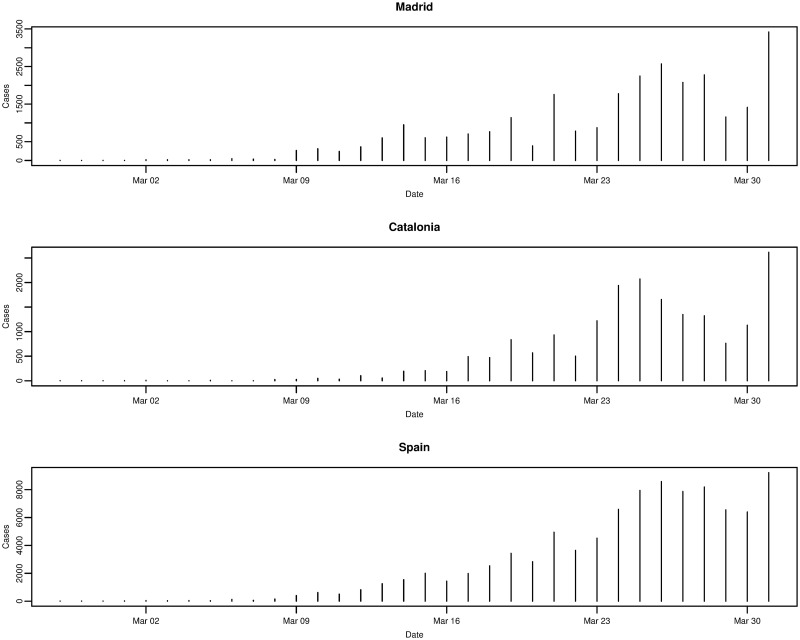
Daily incremental incidence of the Madrid and Catalonia regions, and Spain for the period of 27/02/2020 to 31/03/2020, inclusive.

The worst affected regions in Spain are Madrid and Catalonia, and Fig 8 plots the daily incremental incidence for both regions and the national trend. The growth in daily incidence, in all three cases, could be classed as being approximately exponential, however, daily incidence appears to peak on 26th March 2020 before falling and peaking again on 31st March 2020. It is confirmed that the true peak daily incidence does indeed occur on 31st March 2020 and we return to this point later on in the analysis. In comparison to other Spanish regions, it seems that Madrid and Catalonia are the exceptions as the majority of regions exhibit an exponential rise in daily incidence and peak around 26th and 27th March 2020 before falling.

**Fig 9:**
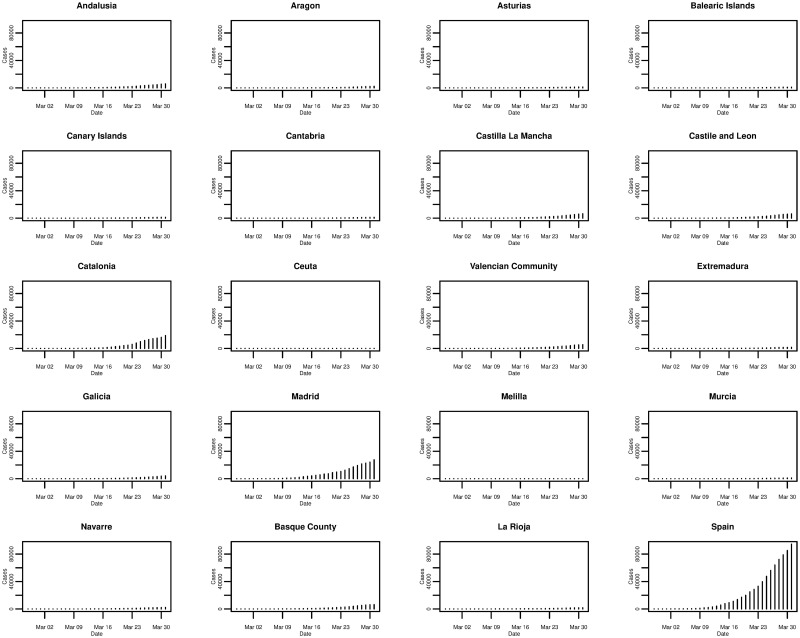
Daily incremental incidence (common scale) of the 19 Spanish regions and Spain for the period of 27/02/2020 to 31/03/2020, inclusive.

Plotting the daily incidence of all regions on the same scale in Fig 9, it is clear that Madrid and Catalonia are the most affected regions contributing the largest share of the national cumulative incidence. Whilst Madrid and Catalonia are the main epicentres of the outbreak in Spain, many coastal regions also show significant numbers of confirmed cases, although not quite on the same scale.

## Method

### The SIR (Susceptible-Infectious-Recovered) model

In the mathematical modelling of infectious diseases, there exist many compartmental models that can be used to describe the spread of a disease within a population. One of the simplest models is the SIR (Susceptible-Infectious-Recovered) model proposed by [46], in which the population is split into three groups or compartments: those who are susceptible (*S*) but not yet infected with the disease; those who are infectious (*I*); those who have recovered (*R*) and are immune to the disease or who have deceased.

The SIR model has been extensively researched and applied in practice, thus it would not be practical to mention and cover all of the literature. However, some of the most prominent literature covers areas such as the stability and optimality of the simple SIR model ([47–51]); pulse vaccination strategy in the SIR model ([52–55]); applications of the SIR in the modelling of infectious diseases ([56–64]).

With regards to COVID-19, many have applied the basic SIR model (or slightly modified versions) to model the outbreak. Some particular examples include (but are not limited to): [2] who estimate the overall symptomatic case fatality risk of COVID-19 in Wuhan and use the SIR model to generate simulations of the COVID-19 outbreak in Wuhan; [65] who apply a modified SIR model to identify contagion, recovery, and death rates of COVID-19 in Italy; [66] who combine the SIR model with probabilistic and statistical methods to estimate the true number of infected individuals in France; [67] who use a number of methods including the SIR model to estimate the basic and controlled reproduction numbers for the COVID-19 outbreak in Wuhan, China; [68] who show that the basic SIR model performs better than extended versions in modelling confirmed cases of COVID-19 and present predictions for cases after the lockdown of Wuhan, China; [69] who model the temporal dynamics of COVID-19 in China, Italy, and France, and find that although the rate of recovery appears to be similar in the three countries, infection and death rates are more variable; [70] who simulate the outbreak in Wuhan, China, using an extended SIR model and investigate the age distribution of cases; [71] who study the number of infections and deaths from COVID-19 in Sweden using the SIR model; [72] who use the SIR model, with an additional parameter for social distancing, to model and forecast the early stages of the COVID-19 outbreak in Brazil.

The SIR model proposed by [46] assumes a fixed population size of *N* and the variables *S*(*t*), *I*(*t*), and *R*(*t*), denote the number of individuals in the three groups mentioned above, as functions of time *t*. Following [73], this model is formed of a system of three differential equations

$$\frac{dS}{dt}=-\frac{\beta IS}{N},\;S\left(0\right)=S_{0}\geq 0,$$ (1)

$$\frac{dI}{dt}=\frac{\beta IS}{N}-\gamma I,\;I\left(0\right)=I_{0}\geq 0,$$ (2)

$$\frac{dR}{dt}=\gamma I,\;R\left(0\right)=R_{0}\geq 0,$$ (3)

where *S*(*t*) + *I*(*t*) + *R*(*t*) = *N*. These equations model the dynamics of the outbreak of an infectious disease and the rates of change in each group. It is assumed that the model uses standard incidence, has a recovery rate of *γI* (Eq (3)), and that the time period under analysis is short enough such that *N* is constant (e.g. there are no births or deaths).

In reference to the SIR model, [74] note that it “examines only the temporal dynamics of the infection cycle and should thus be appropriate for the description of a well-localised epidemic outburst”, therefore, it would appear to be reasonable for use in analysis at city, province, or country level. In the form above, the dynamics of the model are controlled by the parameters *β* and *γ*, representing the rates of transition from *S* to *I* (susceptibility to infection), and *I* to *R* (infection to recovery or death), respectively.

By solving this system of differential equations, it is possible to obtain estimates for the parameters *β* and *γ*. A number of methods can be used to fit the SIR model to incidence data including the least squares method and method of maximum likelihood—in this analysis, the former is chosen. The least squares method focuses on minimising the residual sum of squares—in this particular case, the sum of the squared differences between *I*(*t*) (true number of infected individuals at time *t*) and the predicted number of infected individuals $\hat{I}\left(t\right)$ from the fitted model, expressed as:

$$\begin{array}{c} RSS\left(\beta ,\gamma \right)=\sum_{t=1}^{T}(I\left(t\right)-\hat{I}\left(t\right){)}^{2}, \end{array}$$ (4)

with respect to *β* and *γ*, where *T* denotes the time period up to which the number of infected individuals is accounted for in the model.

To fit the model and find the optimal parameter values of *β* and *γ*, we use the optim function in R [75] to solve the minimisation problem. The system of differential equations, Eqs (1) to (3), are set up as a single function. The model is then initialised with starting values for *S*, *I*, and *R*, with parameters *β* and *γ* unknown. We obtain the daily cumulative incidence for the sample period, total population (*N*), and the susceptible population (*S*) as the total population minus the number of currently infected individuals. This is defined as the cumulative number of infected individuals minus the number of recovered or dead, however, these exact values are difficult to obtain. Thus, the cumulative number of infected individuals at the start date of the sample period is used as a proxy—since at the start date of the disease, this is likely to be close to the true value, as the number of recovered or dead should be very small (if not zero).

The residual sum of squares is then defined and set up as a function of *β* and *γ*. The optim package is used for general purpose optimisation problems, and in this case it is used to minimise the function *RSS* with respect to the sample of cumulative incidence. More specifically, we use the limited memory Broyden-Fletcher-Goldfarb-Shanno (L-BFGS-B) algorithm for the minimisation, which allows us to specify box constraints (lower and upper bounds) for the unknown parameters *β* and *γ*. The lower and upper bounds of zero and one, respectively, were selected for both parameters. The optim function then searches for the *β* and *γ* that minimise the *RSS* function, given starting values of 0.5 for both parameters. The optimal solution is found via the gradient method by repeatedly improving the estimates of *RSS* to try and find a solution with a lower value. The function makes small changes to the parameters in the direction of where *RSS* changes the fastest, where in this direction the lowest value of *RSS* is. This is repeated until no further improvement can be made or the improvement is below a threshold.

We consider convergence as the main criteria for finding an optimal solution in the minimisation of *RSS*—when the lowest *RSS* has been found, and no further improvement can be found or the improvement is below a threshold. In the case where convergence is not achieved, or there is some related error, then we use the parscale function in the optimisation. As the true values of *β* and *γ* are unknown, in the default case, the parameters are adjusted by a fixed step starting from their initial values. Most common issues were addressed using the parscale function to rescale—alter the sensitivity/magnitude of the parameters on the objective function. In other words, it allows the algorithm to compute the gradient at a finer scale (similar to the ndeps parameter—used to adjust step sizes for the finite-difference approximation to the gradient). In most cases, issues were solved by using a step size of 10^−4^. Of course, smaller step sizes could be used, but there is a risk that selecting too small a step size will lead to the optimal values of *β* and *γ* being found at their starting values. However, the results should be interpreted with caution. It is possible that estimates will vary with different population sizes *N* and the starting values specified for *β* and *γ*, which may also cause the optimisation process to be unstable.

It should be noted that the application of the basic SIR model to COVID-19 simplifies the analysis and makes the strong assumption that individuals who become infected but recover are immune to COVID-19. This is assumed purely for the simplification of modelling and we do not claim this to be true in reality. At present, it remains unclear whether those who recover from infection are immune [76]. Indeed, there have been studies and unconfirmed reports of individuals who have possibly recovered but then subsequently tested positive for the virus again, see for example [77–79].

### The basic reproduction number *R*_0_

Whilst the fitted model and optimal parameters allow us to make a simple prediction about how the trajectory of the number of susceptible, infectious, and recovered individuals evolves over time, a more useful statistic or parameter that can be computed from the fitted model is the basic reproduction number *R*_0_. Originally developed for the study of demographics in the early 20th century, it was adapted for use in the study of infectious diseases in the 1950’s [80]. It is defined as the “expected number of secondary infections arising from a single individual during his or her entire infectious period, in a population of susceptibles” [80], and is widely considered to be a fundamental concept in the study of epidemiology. In other words, it is the estimated number of people that an individual will go on to infect after becoming infected.

The *R*_0_ value can provide an indication of the severity of the outbreak of an infectious disease: if *R*_0_ < 1, each infected individual will go on to infect less than one individual (on average) and the disease will die out; if *R*_0_ = 1, each infected individual will go on to infect one individual (on average) and the disease will continue to spread but will be stable; if *R*_0_ > 1, each infected individual will go on to infect more than one individual (on average) and the disease will continue to spread and grow, with the possibility of becoming a pandemic ([80, 81]).

From the basic SIR model above, the reproduction number is defined as

$$\begin{array}{c} R_{0}=\frac{\beta }{\gamma }, \end{array}$$ (5)

and can be estimated by simply replacing *β* and *γ* with their (estimated) optimal values ([73, 81]). Whilst this provides a numerical value indicating the transmissibility of a disease, it should be interpreted with caution due to a number of pitfalls. It is generally assumed that *R*_0_ corresponds to an environment in which there are no individuals who are infected or immune to the disease. This may be more realistic at the beginning of an outbreak, however, outbreaks are rarely observed and modelled at the exact point where infected individuals mix with those who are susceptible. In addition, *R*_0_ values computed under different models can vary, thus the value is dependent on the specific model and its parameters. In particular, *R*_0_ derived from systems of ordinary differential equations (ODEs) can be problematic and may not represent the true *R*_0_ value. Instead, they may simply be representing a threshold value. Furthermore, it is entirely possible for infectious diseases with *R*_0_ < 1 to continue to grow and those with *R*_0_ > 1 to die out [81].

### Log-linear model

**Fig 10:**
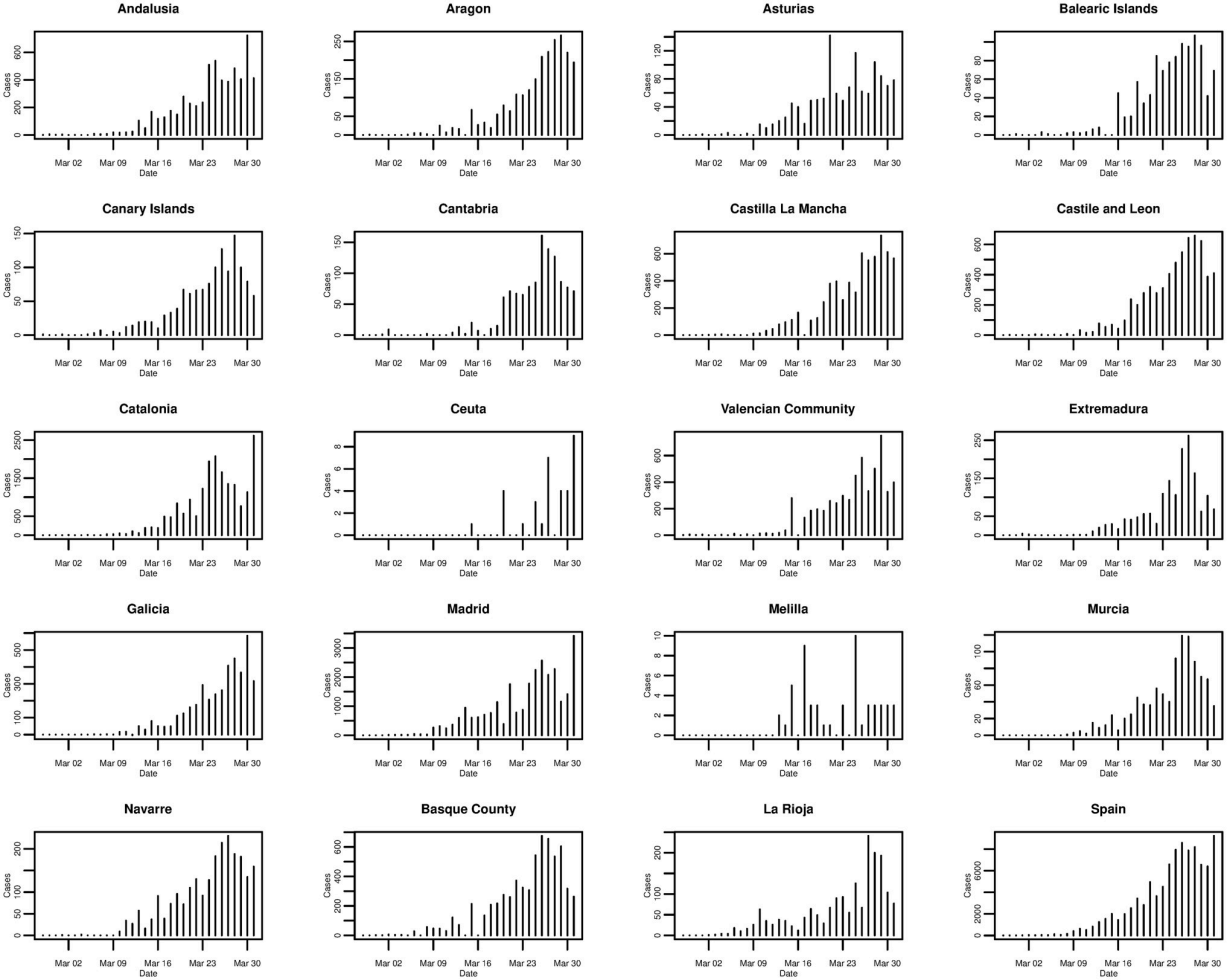
Daily incremental incidence of the 19 Spanish regions and Spain for the period of 27/02/2020 to 31/03/2020, inclusive.

Another simple method to model the incidence of infectious diseases is to use a log-linear (regression) model. The outbreaks of infectious diseases can generally be split into two phases: the growth phase and the decay phase. Given the sample data in this analysis, we focus on the initial growth phase. From Figs 4 and 10 in the data section, it is found that for Italy and Spain (nationally), and their most affected regions, the daily incremental incidence exhibits an approximate exponential trend. It follows that the logarithm of the daily incidence approximately follows a linear trend. In the simplest case, this can be expressed in the form of a simple linear regression

$$\begin{array}{c} \text{log}(y)=b+rt, \end{array}$$ (6)

where *y* denotes the daily incidence, *r* denotes the growth rate, *t* denotes the number of days since the first confirmed cases, and *b* is a constant representing the intercept [82].

To fit the log-linear model, we use the incidence package [82] in R [75] to obtain the optimal values of the parameters. Using the estimated parameters, the fitted model can be used to predict the trajectory of the incidence up until the peak incidence in the growth phase. However, although the log-linear model allows for the modelling and prediction of the incidence, compared with the SIR model it does not provide any indication about the number of susceptible or recovered individuals.

Like with the SIR model, the *R*_0_ value can also be computed using the log-linear model with the key parameter in Eq (6) being the growth rate *r*. [83] show that the growth rate and *R*_0_ are connected by the linear relationship

$$\begin{array}{c} R_{0}=1+\frac{r}{b}, \end{array}$$ (7)

where *r* is the observed (or estimated) exponential growth rate as in Eq (6), and *b* denotes the same rate as *γ* in Eq (3).

We are able to use the epitrix R package [84] to implement the method by [83] for empirical distributions to estimate *R*_0_ from the growth rate *r*. However, [83] note that an “epidemic model implicitly specifies a generation interval distribution” (also known as the serial interval distribution), which is defined as “the time between the onset of symptoms in a primary case and the onset of symptoms in secondary cases” [85]. As we do not have access to more detailed COVID-19 patient data, we are not able to compute the parameters of the serial interval distribution directly. However, a number of existing analyses of COVID-19 patient data report some preliminary estimates of the best fitting serial interval distributions and their corresponding model parameters. These are: i) gamma distribution with mean *μ* = 7.5 and standard deviation *σ* = 3.4 [81]; ii) gamma distribution with mean *μ* = 7 and standard deviation *σ* = 4.5 [2]; iii) gamma distribution with mean *μ* = 6.3 and standard deviation *σ* = 4.2 [86]. By using these three serial intervals in conjunction with the above method, we are able to obtain estimates of *R*_0_ from estimates of the growth rate *r*. It should be noted that serial interval distributions are not only restricted to the gamma distribution—other common distributions used include the Weibull and log-normal distributions, and that the parameters are dependent on a number of factors including the time to isolation [86].

### The effective reproduction number *R*_*e*_

As mentioned above, the estimation of the *R*_0_ value is not always ideal, due to it being a single fixed value reflecting a specific period of growth (in the log-linear model) or requiring assumptions that only hold true in specific time periods (in the basic SIR model). In other words, it is “time and situation specific” [85]. In reality, the reproduction number will vary over time but it will also be influenced by governments and health authorities implementing measures in order to reduce the impact of the disease. Therefore, a more useful approach for measuring the severity of an infectious disease is to track the reproduction number over time. The effective reproduction number *R*_*e*_ is one way to achieve this, and thus allows us to see how the reproduction number changes over time in response to the development of the disease itself but also effectiveness of interventions. Although there are numerous methods that can be used to analyse the severity of a disease over time, the majority are not straightforward to implement (especially in software) [85].

One popular method for estimating *R*_*e*_ is that proposed by [85]. The basic premise of this method is that “once infected, individuals have an infectivity profile given by a probability distribution *w*_*s*_, dependent on time since infection of the case, *s*, but independent of calendar time, *t*. For example, an individual will be most infectious at time *s* when *w*_*s*_ is the largest. The distribution *w*_*s*_ typically depends on individual biological factors such as pathogen shedding or symptom severity” [85].

The instantaneous (or effective) reproduction number *R*_*e*_ at true time *t*, can be estimated by the ratio of the number of new infections occurring at time *t*, denoted by *I*_*t*_, to the total infectiousness of infected individuals at time *t*—the sum of the weighted daily incidence up to time *t* − 1 weighted by infectivity, $\sum_{s=1}^{t}I_{t-s}w_{s}$. We implement the method by [85] in R [75] using the EpiEstim package [87]. We use the daily incidence and corresponding dates in combination with the three serial intervals (their means and standard deviations) in the estimation function in the EpiEstim package [87]. We use the parametric_si method, as we do not have have access to patient level data to estimate the serial interval distributions. This method computes the discrete serial interval assuming a gamma distribution given specified values for the mean and standard deviation—conveniently, the three sets of serial intervals from the literature correspond with the gamma distribution.

The function models the transmissibility of a disease with a Poisson process, such that an individual infected at time *t* − *s* will generate new infections at time *t* at a rate of *R*_*t*_ *w*_*s*_, where *R*_*t*_ is the instantaneous (effective) reproduction number at time *t*. Thus, the incidence at time *t* is defined to be Poisson distributed with mean equal to the average daily incidence (number of new cases) at time *t*. This value is just for a single time period *t*, however, estimates for a single time period can be highly variable meaning that it is not easy to interpret, especially for making policy decisions. Therefore, we consider longer time periods of one week (seven days)—assuming that within a rolling window the instantaneous reproduction number remains constant. Note that there is a potential trade off, as using longer rolling windows gives more precise estimates of *R*_*t*_ but this means fewer estimates can be computed (requires more incidence values to start with) and a more delayed trend reducing the ability to detect changes in transmissibility. Whereas shorter rolling windows lead to more rapid detection in changes but with more noise. Using this method, it is recommended that a minimum cumulative daily incidence of 12 cases have been observed before attempting to estimate *R*_*e*_. For the data sets used, this does not pose a problem as a cumulative total of 16 and 17 cases, respectively, exist on the first day of the sample at the country level, and by the seventh day the totals are around 200 and 650 for Spain and Italy, respectively.

Using Bayesian statistical inference based on the transmission model, the function computes the posterior distribution of *R*_*t*_ under the assumption that *R*_*t*_ is gamma distributed, with parameters

$$\begin{array}{c} a+\sum_{s=t-\tau +1}^{t}I_{s} \end{array}$$

and

$$\begin{array}{c} \frac{1}{\frac{1}{b}\sum_{s=t-\tau +1}^{t}\Lambda_{s}}, \end{array}$$

respectively, where $\Lambda_{t}=\sum_{s=1}^{t}I_{t-s}w_{s}$.

From the posterior distribution, the posterior mean *R*_*t*,*τ*_ can be computed at time *t* for the rolling window of [*t* − *τ*, *t*] by the ratio of the gamma distribution parameters. We refer the readers to the supplementary information of [85] for further details regarding the Bayesian framework. As noted by [85], this method works best when times of infection are known and the infectivity profile or distribution can be estimated from patient level data. However, as mentioned above, we do not have access to this level of data, and instead utilise three different serial intervals from the literature that have been estimated from real data.

In practice, the transmission of a disease will vary over time especially when health prevention measures are implemented. However, this method is the only reproduction number that can be easily computed in real-time, and in comparison to similar methods, it captures the effect of control measures since it will cause sudden decreases in estimates compared with other methods.

In this analysis, we use the most basic version of this method and estimate the effective reproduction number over a rolling window of seven days. This appears to be sufficient and in line with our results, as we do not suffer from the problem of small sample sizes as the samples are sufficiently large and we start computing the effective reproduction number after one mean serial interval. It should be noted that estimates of this reproduction number are dependent on the distribution of the infectiousness profile *w*_*s*_. In addition, it is known that this distribution may not always be well documented, especially in the early parts of an epidemic. However, here we assume that the serial interval is defined for our sample period and the use of the three serial intervals from the literature appears to give satisfactory results.

If problems did arise, or to account for uncertainty in the serial interval distribution, an alternative method is to implement a modified procedure by [85], which allows for uncertainty in the serial interval distribution. This modified method assumes that the serial interval is gamma distributed but the mean and standard deviation are allowed to vary according to a standard normal distribution. Some N* pairs of means and standard deviations are simulated—mean first and standard deviation second, with the constraint that the mean is less than the standard deviation to ensure that for each pair the probability density function of the serial interval distribution is null at time *t* = 0. Then, for each rolling window 1000 realisations are sampled of the instantaneous reproduction number using the posterior distribution conditional on the pair of parameters.

## Results

### The SIR model and *R*_0_

For both Italy and Spain, we set up and solve the minimisation problem for the SIR model described in Section for region-level and national-level COVID-19 incidence for the first 14 days after the first cases were confirmed in each respective country and region. The first 14 days after the first cases are detected can be considered to be the early stage of an outbreak, and it is reasonable to assume that there are few, if no, infected or immune individuals prior to this. However, it is a rather strong assumption as it is possible that individuals may be infected but do not display any symptoms. Tables 1 and 2 show the output corresponding to each region/country including the date that the first cases were confirmed, the population size (obtained from [88]), the cumulative number of cases at the 14th day after the first cases were confirmed, the fitted estimates for the parameters *β* and *γ*, and estimates for *R*_0_.

**Table 1:** The estimated SIR model parameters and *R*_0_ values for Italy and its regions, in the 14 days after the first confirmed cases.

Region	Date of First Case	Population	Cumulative Cases	$\hat{\beta }$	$\hat{\gamma }$	${\hat{R}}_{0}$
Lombardy	21-Feb-2020	10,060,000	2,251	1.000	0.602	1.660
Veneto	21-Feb-2020	4,906,000	407	0.714	0.286	2.493
Emilia-Romagna	22-Feb-2020	4,459,000	870	0.740	0.260	2.846
Piedmont	22-Feb-2020	4,356,000	145	0.695	0.305	2.277
Sicily	25-Feb-2020	5,000,000	56	0.614	0.386	1.593
Tuscany	25-Feb-2020	3,730,000	208	0.681	0.319	2.134
Liguria	25-Feb-2020	1,551,000	132	1.000	0.616	1.623
South Tyrol	25-Feb-2020	520,891	9	0.583	0.417	1.397
Marche	26-Feb-2020	1,525,000	394	0.737	0.263	2.801
Campania	27-Feb-2020	5,802,000	154	0.657	0.343	1.918
Apulia	27-Feb-2020	4,029,000	77	1.000	0.659	1.517
Abruzzo	27-Feb-2020	1,312,000	38	0.652	0.362	1.802
Calabria	28-Feb-2020	1,947,000	33	0.255	0.000	Inf
Lazio	29-Feb-2020	5,879,000	274	0.684	0.333	2.055
Friuli-Venezia Giulia	01-Mar-2020	1,215,000	301	0.660	0.354	1.864
Umbria	01-Mar-2020	882,015	107	0.654	0.346	1.891
Sardinia	03-Mar-2020	1,640,000	107	0.681	0.319	2.135
Basilicata	03-Mar-2020	562,869	12	0.608	0.402	1.511
Trentino	03-Mar-2020	538,223	378	1.000	0.640	1.564
Molise	03-Mar-2020	305,617	21	0.581	0.419	1.386
Aosta Valley	05-Mar-2020	125,666	165	0.673	0.327	2.054
**Italy**	21-Feb-2020	**60,360,000**	**3,855**	**0.715**	**0.285**	**2.505**

**Table 2:** The estimated SIR model parameters and *R*_0_ values for Spain and its regions, in the 14 days after the first confirmed cases.

Region	Date of First Case	Population	Cumulative Cases	$\hat{\beta }$	$\hat{\gamma }$	${\hat{R}}_{0}$
Andalusia	27-Feb-2020	8,427,000	91	0.676	0.324	2.090
Catalonia	27-Feb-2020	7,566,000	156	0.668	0.332	2.009
Madrid	27-Feb-2020	6,662,000	1024	1.000	0.570	1.753
Valencia Community	27-Feb-2020	4,795,000	65	0.636	0.364	1.750
Canary Islands	27-Feb-2020	2,153,000	33	0.631	0.369	1.713
Castile and Leon	28-Feb-2020	2,408,000	92	0.647	0.353	1.829
Aragon	28-Feb-2020	1,321,000	65	0.659	0.341	1.936
Basque Country	29-Feb-2020	2,178,000	417	0.710	0.290	2.450
Balearic Islands	29-Feb-2020	1,188,000	29	0.255	0.000	Inf
Castilla-La Mancha	01-Mar-2020	2,035,000	289	0.436	0.000	Inf
Extremadura	01-Mar-2020	1,065,000	66	0.592	0.408	1.453
Asturias	01-Mar-2020	1,022,000	92	0.682	0.344	2.041
Navarre	01-Mar-2020	649,946	146	0.694	0.306	2.269
Cantabria	01-Mar-2020	581,641	31	0.635	0.365	1.738
La Rioja	02-Mar-2020	315,675	300	1.000	0.540	1.850
Galicia	04-Mar-2020	2,700,000	292	0.735	0.287	2.563
Murcia	08-Mar-2020	1,488,000	240	1.000	0.564	1.772
Melilla	13-Mar-2020	84,689	39	1.000	0.755	1.325
Ceuta	15-Mar-2020	84,829	17	0.611	0.389	1.571
**Spain**	27-Feb-2020	**46,940,000**	**2,128**	**0.698**	**0.320**	**2.180**

From Tables 1 and 2, we observe that many of the first regions to be affected in both countries are those with the largest population sizes, however, the cumulative number of cases (after the first 14 days) in these regions are not always the highest among all regions. The estimates of the parameters *β* and *γ* also do not show any particular trends and this is reflected in the estimated *R*_0_ values. It can be seen that for all regions in both Italy and Spain, the estimated *R*_0_ values fall between one and three. This suggests that, according to the thresholds described above, the disease is spreading and growing in all Italian and Spanish regions during the 14 days after the first localised cases were confirmed. At a national level, the estimated values of *R*_0_ are greater than two for both countries, again, suggesting a spreading and growing disease. This is perhaps not surprising since this time period reflects the early stages of the spread of the disease, thus we would expect it to be growing and spreading quickly before any preventative action is taken.

We note that in Tables 1 and 2, there are some cases where the estimated value of *β* is very close to or at the upper limit of 1.000—e.g. Lombardy (Italy) and Madrid (Spain). This leads to the consequence that the parameter estimates appear to be bound by the upper limit. However, all parameter estimates are dependent on the starting values defined for *β* and *γ*, and the upper and lower bounds specified. For all cases of estimating the parameters in Tables 1 and 2, we used the same optimisation procedure and criteria for determining a satisfactory estimate that is the convergence in the minimisation of the *RSS* (Eq (4)). In all cases, convergence was achieved but this is still slightly problematic. For cases where the estimated value of *β* is 1.000, although convergence was achieved, this indicates only that it generates the lowest RSS within the upper and lower limits defined. Therefore, there may or may not exist values of the parameter outside of this range that may be more optimal. Indeed, the results may vary depending on the upper and lower bounds, and the starting values that are selected. Thus, there is also the question of how to change the starting values and bounds appropriately (instead of, say, simply increasing them). Furthermore, as the *R*_0_ value in the SIR model is computed as *β*/*γ*, another consequence of the estimated value of *β* being 1.000 is that the true value of *β* may actually be larger than this, and so the true value of *R*_0_ may be larger than the estimated value.

**Fig 11:**
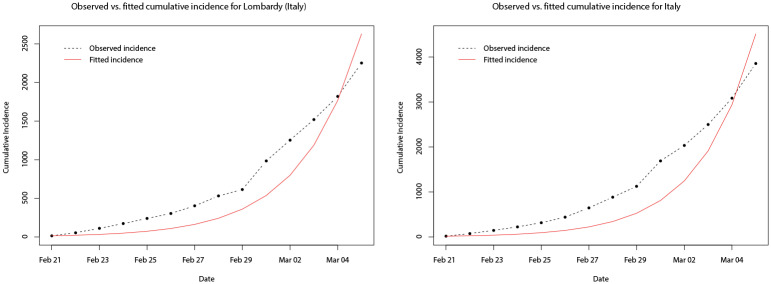
Plots of the observed (dot-dashed black line) and fitted (solid red line) cumulative incidence for Lombardy and Italy, for the 14 days after the first confirmed cases.

**Fig 12:**
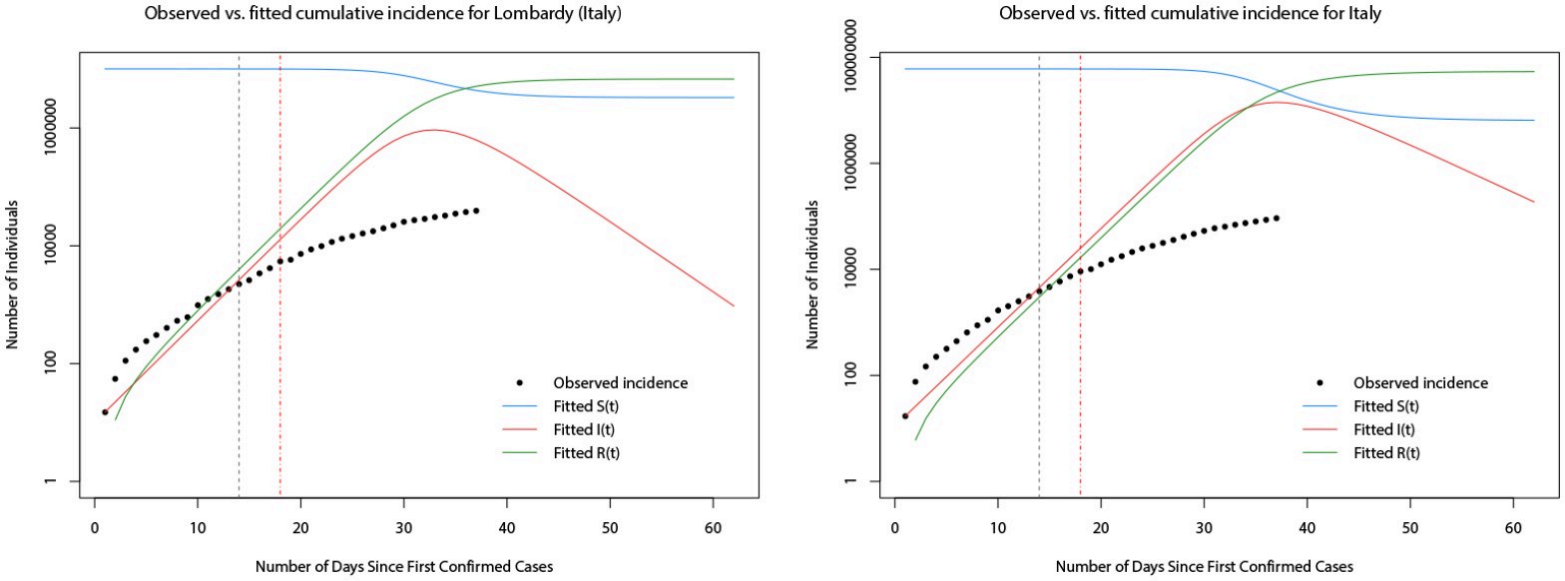
Plots of the observed cumulative incidence (solid black points) for Lombardy and Italy, and the fitted values of *S*(*t*) (solid blue line), *I*(*t*) (solid red line), and *R*(*t*) (solid green line) for the two months after the first confirmed cases.

Using the estimated parameters for the best fitted models, the predicted trajectories of the numbers in each of the compartments of the model can be generated. For brevity, in the remainder of the analysis, we show only the results for Italy, Spain, and their worst affected regions. Fig 11 plots the observed and predicted cumulative incidence for the 14 days immediately following the first confirmed cases in Lombardy and Italy, respectively. It can be seen that the model appears to under predict the true total number of cases in both cases during the early part of the outbreak before over estimating towards the end of the 14 days. In Fig 12 the SIR model trajectories are plotted along with the observed cumulative incidence on a logarithmic scale for Lombardy and Italy. The under prediction of the cumulative incidence in the first 14 days (to the left of the vertical dashed black line) is indicated by the solid red line (predicted cumulative incidence) lying below the black points (observed cumulative incidence) however, after the initial 14 days and after the implementation of a nationwide lock down (vertical dashed red line), the observed cumulative incidence grows at a slower rate than predicted by the fitted model. Indeed, this reflects the fact that the model is based only on the initial 14 days and does not account for any interventions.

**Fig 13:**
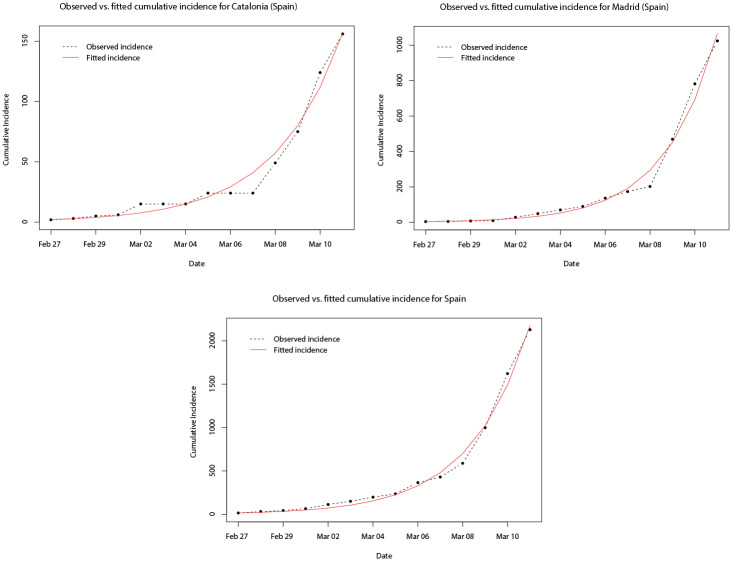
Plots of the observed (dot-dashed black line) and fitted (solid red line) cumulative incidence for Madrid, Catalonia, and Spain, for the 14 days after the first confirmed cases.

**Fig 14:**
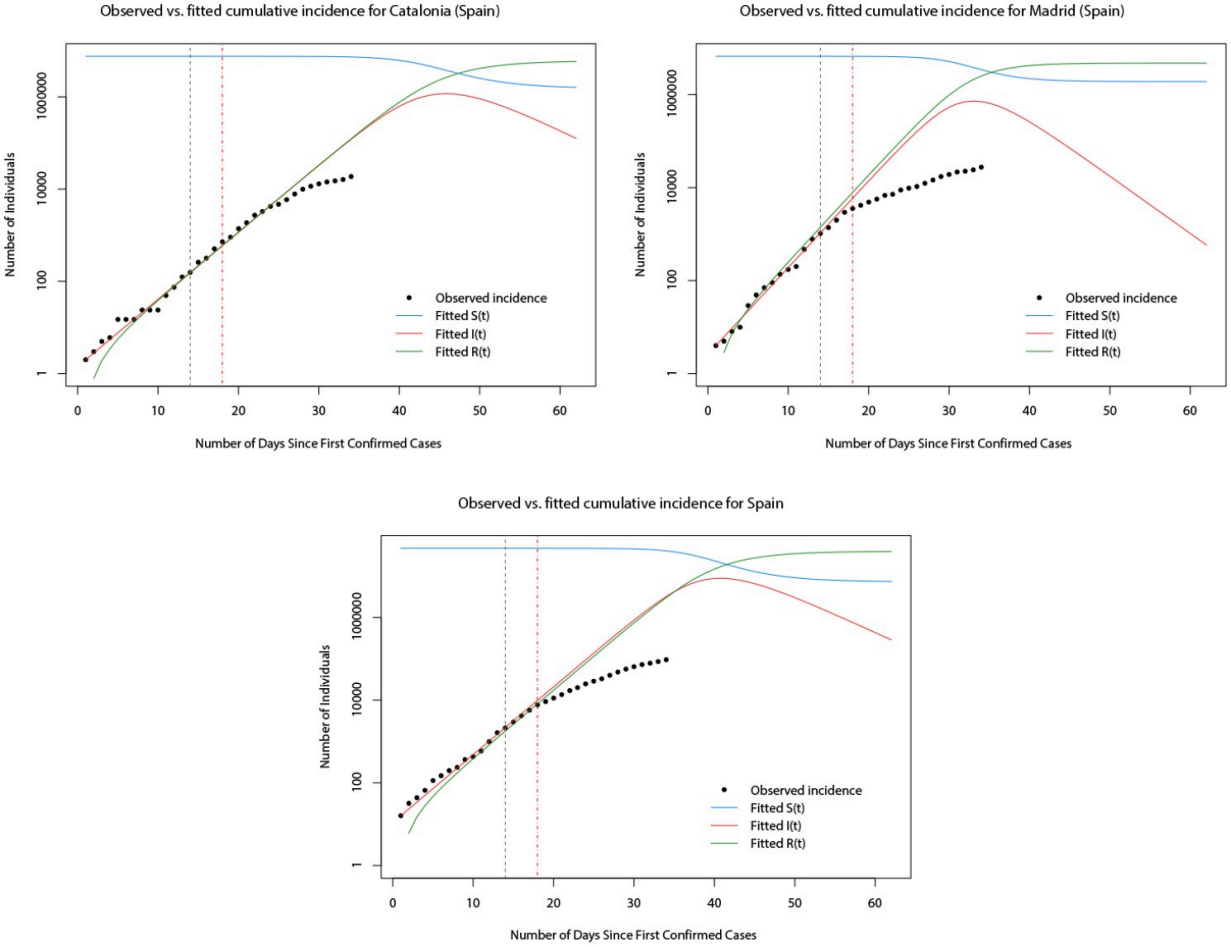
Plots of the observed cumulative incidence (solid black points) for Madrid, Catalonia, and Spain, and the fitted values of *S*(*t*) (solid blue line), *I*(*t*) (solid red line), and *R*(*t*) (solid green line) for the two months after the first confirmed cases.

In Fig 13, the observed and predicted cumulative incidence for the 14 days immediately following the first confirmed cases in Catalonia, Madrid, and Italy, respectively, are shown. In contrast to the results for Italy, the fitted model for all three appears to predict the true total number of cases across the whole of the first 14 days reasonably well. Fig 14 plots the SIR model trajectories and the observed cumulative incidence on a logarithmic scale for Catalonia, Madrid, and Spain. Here, the more accurate predictions of the cumulative incidence are reflected in the area to the left of the vertical dashed black line. However, it can be seen that at the time when the nationwide lock down came into force (vertical dashed red line) the growth of the true total number of cases slowed down. It is likely that this is coincidental, since it is known that the effect on the incidence of infectious diseases from health interventions is not immediate, but instead lags behind.

### Log-linear model and *R*_0_

**Fig 15:**
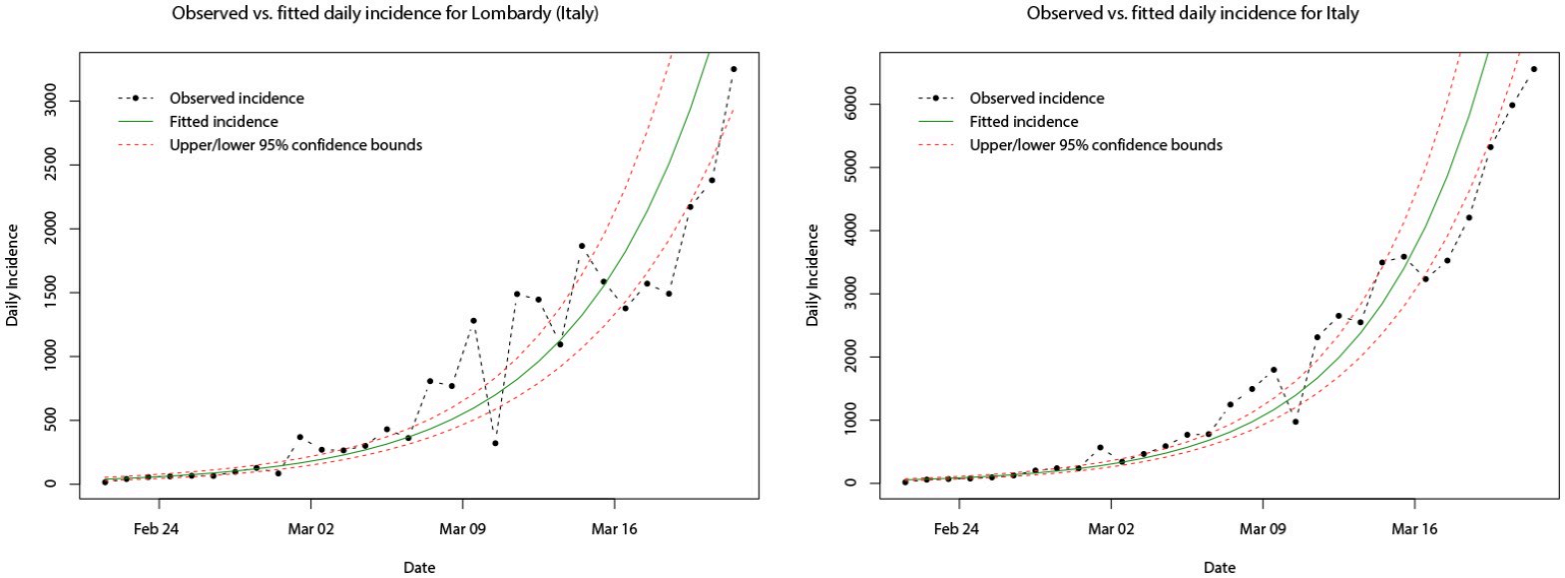
Plots of the observed cumulative incidence (dot-dashed black line) for Lombardy and Italy, and the fitted log-linear model (solid green line) for their respective growth phases. Upper and lower limits of the 95% confidence intervals are indicated by the dashed red lines.

Following the SIR model, we implemented the log-linear model as described above for region-level and national-level COVID-19 daily incidence for the entire growth phase (from the time of the first confirmed cases until the time at which daily incidence peaks). The estimated parameters of the fitted log-linear models for the daily incidence of Lombardy and Italy, respectively, are shown in Table 3. It can be seen that the peak daily incidence in both Lombardy and at country level occurred on the same day (21st March 2020), however, the growth rate (doubling time) is found to be slightly greater (shorter) at country level (0.18 and 3.88) compared with the Lombardy region (0.16 and 4.34). In comparison to the SIR model and modelling the cumulative incidence, the log-linear model modelling the daily incidence in the growth phase (as shown in Fig 15) appears to be slightly more accurate.

**Table 3:** Estimates of the growth rate and doubling time during the growth phase in Lombardy and Italy.

Location	Date of Peak	Growth Rate	Doubling Time
Lombardy	21-Mar-2020	0.160 (0.141, 0.178)	4.342 (3.884, 4.924)
Italy	21-Mar-2020	0.179 (0.163, 0.195)	3.882 (3.563, 4.264)

Upper and lower limits of the 95% confidence intervals are given in parentheses under the estimated values.

**Fig 16:**
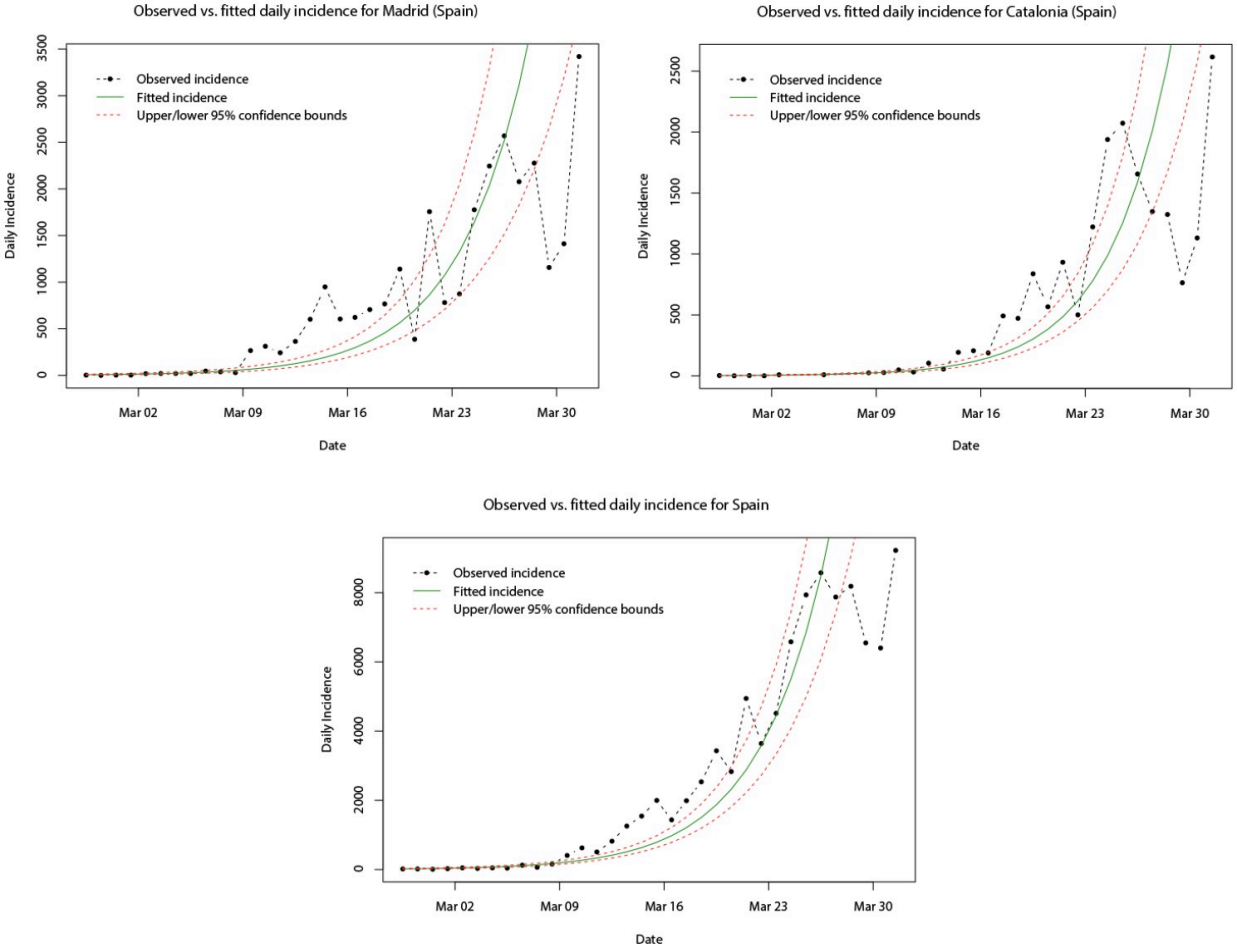
Plots of the observed cumulative incidence (dot-dashed black line) for Madrid, Catalonia, and Spain, and the fitted log-linear model (solid green line) for their respective growth phases. Upper and lower limits of the 95% confidence intervals are indicated by the dashed red lines.

In Table 4, the estimated parameters of the fitted log-linear models for the daily incidence of Madrid, Catalonia, and Spain, respectively, are given. Similarly, the peak daily incidence occurs on the same day (31st March 2020) for Madrid, Catalonia, and Spain, although this is later than that for Italy. Interestingly, the growth rate (doubling time) is greatest (shortest) for Catalonia (0.24 and 3.85), whilst Madrid and Spain share similar growth rates and doubling times (0.21/0.22 and 3.24/3.21). It should be noted that there appears to be a slight difference in the observed daily incidence compared with the case of Italy and its regions. In Fig 16, it can be seen that the observed daily incidence appears to initially peak in the last few days of March in all cases before falling, but then increases to a higher peak at the end of the growth phase. This seems to throw off the fitted log-linear model, as after the initial (approximate) 14 days the fitted model under predicts and then over predicts the daily incidence.

**Table 4:** Estimates of the growth rate and doubling time during the growth phase in Madrid, Catalonia, and Spain.

Location	Date of Peak	Growth Rate	Doubling Time
Madrid	31-Mar-2020	0.214 (0.180, 0.247)	3.243 (2.803, 3.849)
Catalonia	31-Mar-2020	0.237 (0.210, 0.264)	2.920 (2.621, 3.296)
Spain	31-Mar-2020	0.216 (0.194, 0.238)	3.206 (2.909, 3.571)

Upper and lower limits of the 95% confidence intervals are given in parentheses under the estimated values.

As with the SIR model, we are also able to use the fitted log-linear models in conjunction with the three serial intervals mentioned above to compute estimates of the *R*_0_ value. Table 5 shows the mean estimates of the *R*_0_ value for Italy, Spain, and their most affected regions, computed from the fitted log-linear models and the three serial intervals. In each case, the mean estimates are computed from 10,000 samples of *R*_0_ values generated from the log-linear regression of the incidence data in the growth phase, and the distributions of these samples are plotted in S1 Fig. Compared with the estimates from the SIR model, we find that in all but the case of Italy, the estimates of *R*_0_ from the log-linear model are greater than that from the SIR model—in these cases, the lowest estimates of *R*_0_ from the log-linear models are larger by between 0.5 to 1. In the case of Italy, we find that the estimate of *R*_0_ computed from the SIR model is approximately the same as that computed from the log-linear model using a serial interval using a gamma distribution with mean *μ* = 7 and standard deviation *σ* = 4.5 [2]. Using the log-linear models, the largest *R*_0_ values computed are for Catalonia, whereas the smallest values are for Lombardy. It can also be seen that serial distributions with a lower mean appear to correspond with lower *R*_0_ values. A possible explanation for the difference between the estimated *R*_0_ values computed from the SIR models and the log-linear models is that the only incidence data from the first 14 days was used in the former, whereas incidence data from the whole growth phase was used in the latter—almost double the data. Therefore, it is arguable that the *R*_0_ estimates from the log-linear models could be considered to be more accurate.

**Table 5:** Estimates of the *R*_0_ value for Italy, Spain, and their most affected regions during their respective growth phases.

Location	${\hat{R}}_{0}$ (*SI*_1_)	${\hat{R}}_{0}$ (*SI*_2_)	${\hat{R}}_{0}$ (*SI*_3_)
Lombardy	2.689 (2.412, 2.977)	2.313 (2.130, 2.498)	2.121 (1.967, 2.279)
Italy	2.979 (2.724, 3.241)	2.504 (2.342, 2.667)	2.278 (2.143, 2.416)
Madrid	3.582 (2.986, 4.234)	2.877 (2.523, 3.249)	2.582 (2.286, 2.892)
Catalonia	4.035 (3.502, 4.599)	3.143 (2.841, 3.451)	2.795 (2.548, 3.052)
Spain	3.626 (3.222, 4.049)	2.904 (2.668, 3.147)	2.604 (2.407, 2.806)

Assuming serial interval distributions following a gamma distribution with parameters: i) *μ* = 7.5 and *σ* = 3.4 (*SI*_1_); ii) *μ* = 7 and *σ* = 4.5 (*SI*_2_); iii) *μ* = 6.3 and *σ* = 4.2 (*SI*_3_).

#### Effective reproductive number *R*_*e*_

**Fig 17:**
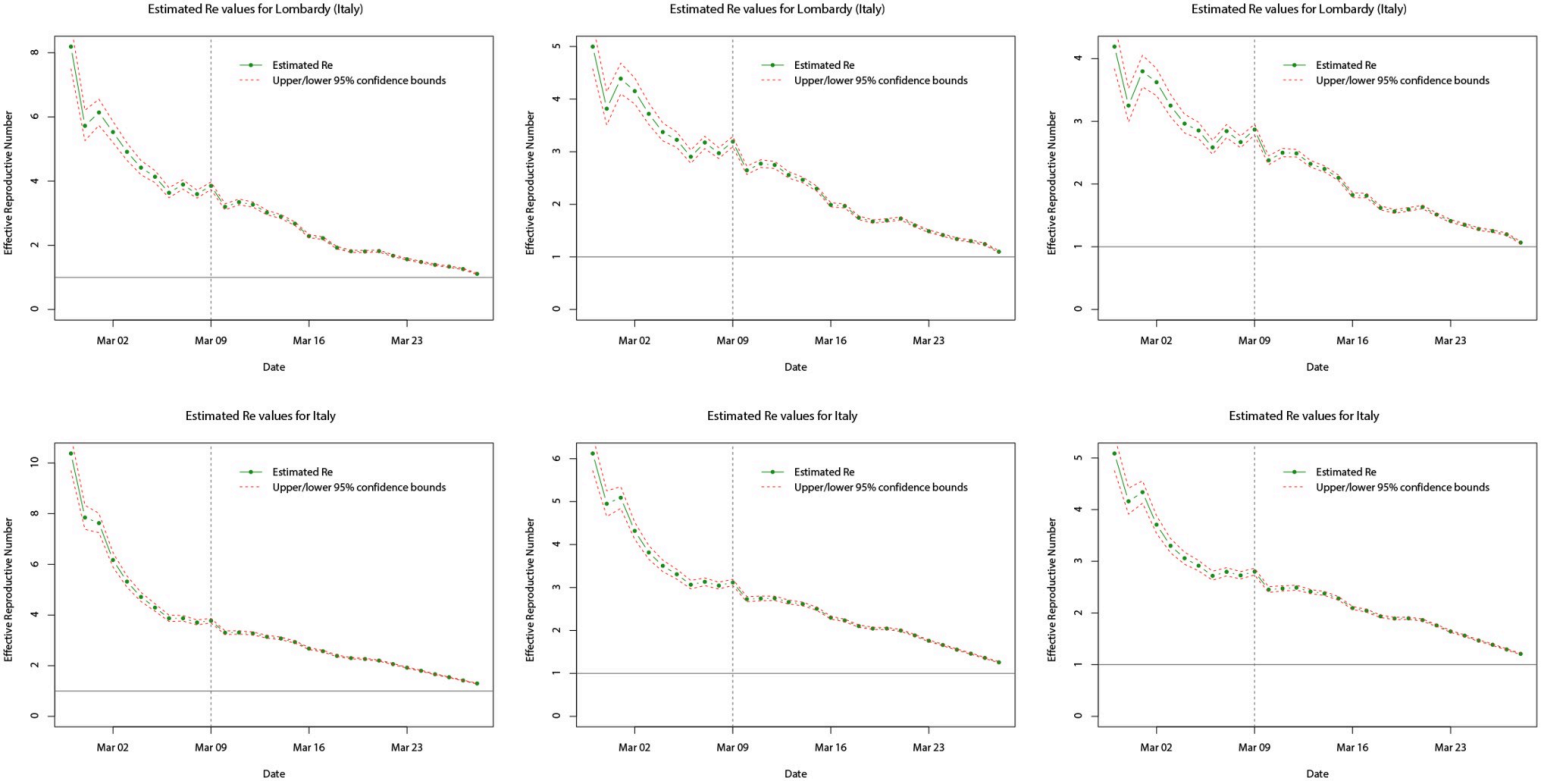
Plots of the estimated mean *R*_*e*_ values (dot-dashed green line) for Lombardy (top row) and Italy (bottom row) over the whole sample period, using serial interval distributions *SI*_1_ (left), *SI*_2_ (middle), and *SI*_3_ (right). Upper and lower limits of the 95% confidence intervals for the mean are indicated by the red dashed lines, and the grey dotted line indicates the date at which the national lock down becomes effective.

**Fig 18:**
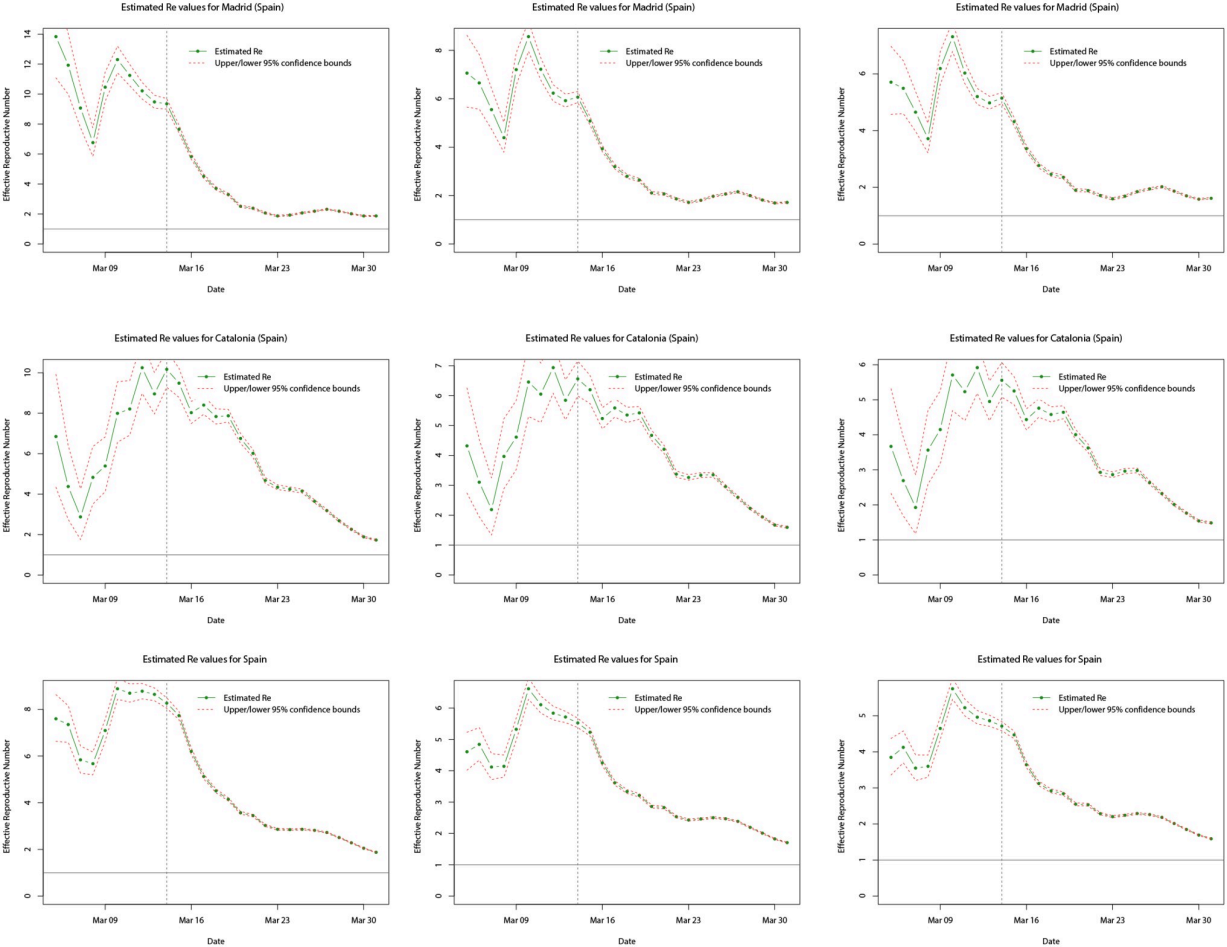
Plots of the estimated mean *R*_*e*_ values (dot-dashed green line) for Madrid (top row), Catalonia (middle row), and Spain (bottom row) over the whole sample period, using serial interval distributions *SI*_1_ (left), *SI*_2_ (middle), and *SI*_3_ (right). Upper and lower limits of the 95% confidence intervals for the mean are indicated by the red dashed lines, and the grey dotted line indicates the date at which the national lock down becomes effective.

Turning towards the more dynamic measure of the infectiousness of diseases, Figs 17 and 18 plot the estimated reproductive numbers computed for Lombardy, Italy, Madrid, Catalonia, and Spain, over the entire sample period. Using the method proposed by [85], in each case estimates were computed using rolling windows of the daily incidence over the previous 7 days and the same three serial distributions as for the log-linear models. As a result, no estimates are computed for the first 7 days of each respective sample period. In all cases, we analyse and compute the *R*_*e*_ values over the whole sample period available allowing us to see how the infectiousness of COVID-19 varies during the initial outbreak stages and the effect of any interventions implemented by the respective governments. In Fig 17, we observe that for both Lombardy and Italy, *R*_*e*_ is generally decreasing over the time (under any of the three serial distributions), and although it is initially larger for Italy, after approximately the first 7 days the *R*_*e*_ values are similar. However, the trend of *R*_*e*_ both to the left and right (before and after) of the nationwide lockdown (indicated by the dotted line) shows some differences. Prior to the nationwide lockdown, *R*_*e*_ decreases rapidly towards a value of between three and four, which could be attributed to the fact that northern Italy (including Lombardy) was the most affected area in the early stages of the outbreak and lockdowns local to the area were already being enforced from 21st February 2020. Thus, this is likely to have contributed (in part) to the initial reduction in the *R*_*e*_ value. After the nationwide lockdown came into force on 9th March 2020, *R*_*e*_ continues to decrease but at a slower pace and appears to level off approximately 14 days later—this coincides with the peak in daily incidence on 21st March 2020. After this point, it is likely that the effects of the nationwide lockdown are starting to appear with *R*_*e*_ appearing to decrease again more rapidly towards the critical value of one (solid horizontal line)—suggesting that the disease is still spreading but stabilising.

In Fig 18, we observe a different trend in the *R*_*e*_ value for Madrid, Catalonia, and Spain, compared with Lombardy and Italy. Whilst *R*_*e*_ exhibits a decrease over the sample time period (under any of the three serial distributions), the initial values are actually larger for Madrid and Catalonia, however, the values for all three are similar after the initial 7 days. The trend in the estimated *R*_*e*_ values before and after the nationwide lockdown again show some differences, but also differ to those for the cases of Lombardy and Italy. Prior to the nationwide lockdown (indicated by the dotted line), the trend of the estimated *R*_*e*_ values is very erratic: decreasing, increasing, and then decreasing again. This could be due to the daily incidence for Madrid, Catalonia, and Spain, showing greater variation compared with that for Italy before the respective lockdowns. It is found that in the period before the lockdowns, Spanish daily incidence appears to show more alternation between increases and decreases compared with the previous day’s incidence, whilst Italian daily incidence shows much less. After the nationwide lockdown on 14th March 2020, for all three cases the estimated *R*_*e*_ decreases significantly towards a value of two. More specifically, in mid-March 2020 daily incidence for Madrid, Catalonia, and Spain, levels off corresponding to the reduction in *R*_*e*_, but in the run up to 23rd March 2020 daily incidence again becomes more variable and alternates between significantly larger and smaller daily incidence, with *R*_*e*_ levelling off. After 23rd March 2020, this levelling off is more sustained for Madrid and Spain compared with Catalonia. This may be attributed to the daily incidence initially peaking and then decreasing much more significantly for Catalonia, leading to a more significant decrease in *R*_*e*_ at the latter end of the sample period. In general, the estimated *R*_*e*_ values are larger for Spain than Italy, since Spain is lagging behind in terms of the start of the outbreak, however, it is found that the estimated *R*_*e*_ is larger for Italy than Spain, but larger for Madrid and Catalonia than Lombardy.

#### Predictive ability of models

Whilst the results regarding the estimated reproduction values (*R*_0_ and *R*_*e*_) provide useful indicators about the infectiousness of COVID-19 and the variability over time, the predictive ability of models is also key—especially in the decay phase of an outbreak after the daily incidence has peaked and is in decline. Predictions about the daily incidence in the decay phase can contribute to determining whether health interventions are working, but can additionally provide time frames for when daily incidence may reach certain thresholds—e.g. below which the disease may be considered under control. To compare the predictive ability of the SIR and log-linear models, we use the projections package [89] in R [75]. As this section acts to provide only a brief analysis of the predictive ability of the models, we refer the readers to [89] for in-depth documentation regarding the finer details of the computations. The initial step is to consider which of the two models provides the best predictive ability in the growth phase of the COVID-19 outbreak and for simplicity, we analyse only Italy and Spain at country level. Using the estimated *R*_0_ values for Italy and Spain from the SIR and log-linear models above, we combine these with the three serial distributions mentioned earlier. We then use the projections package [89] to forecast and predict the daily incidence for Italy and Spain from the 14th day (since the first cases in each location) until the day of peak incidence.

**Fig 19:**
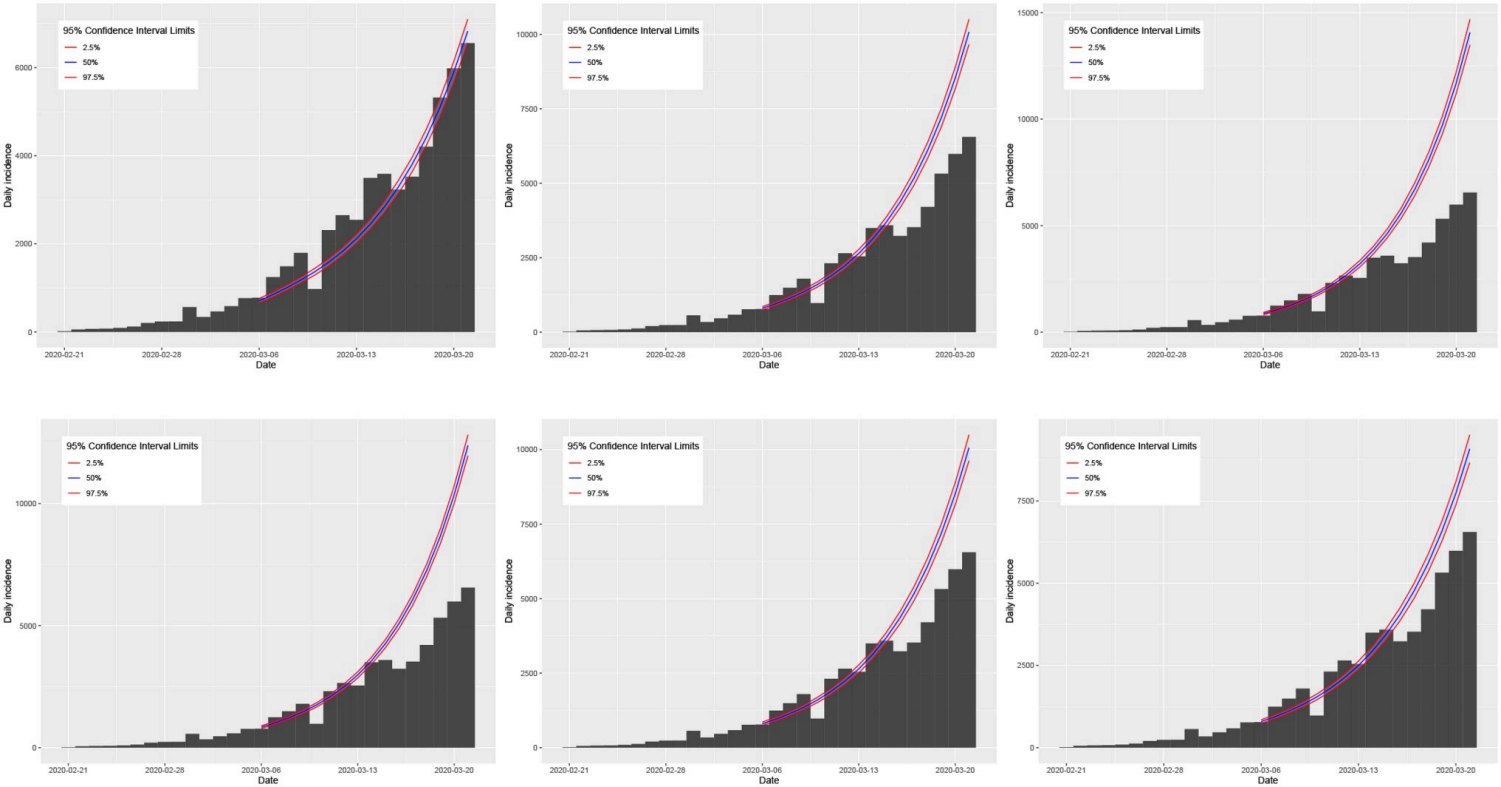
Plots of the observed daily incidence (histograms) in Italy during its growth phase and the predicted daily incidence (purple solid line) estimated using the SIR model (top row) and log-linear model (bottom row) assuming serial interval distributions of *SI*_1_ (left), *SI*_2_ (middle), and *SI*_3_ (right). 95% confidence intervals for the predicted incidence are indicated by the shaded light purple regions.

**Fig 20:**
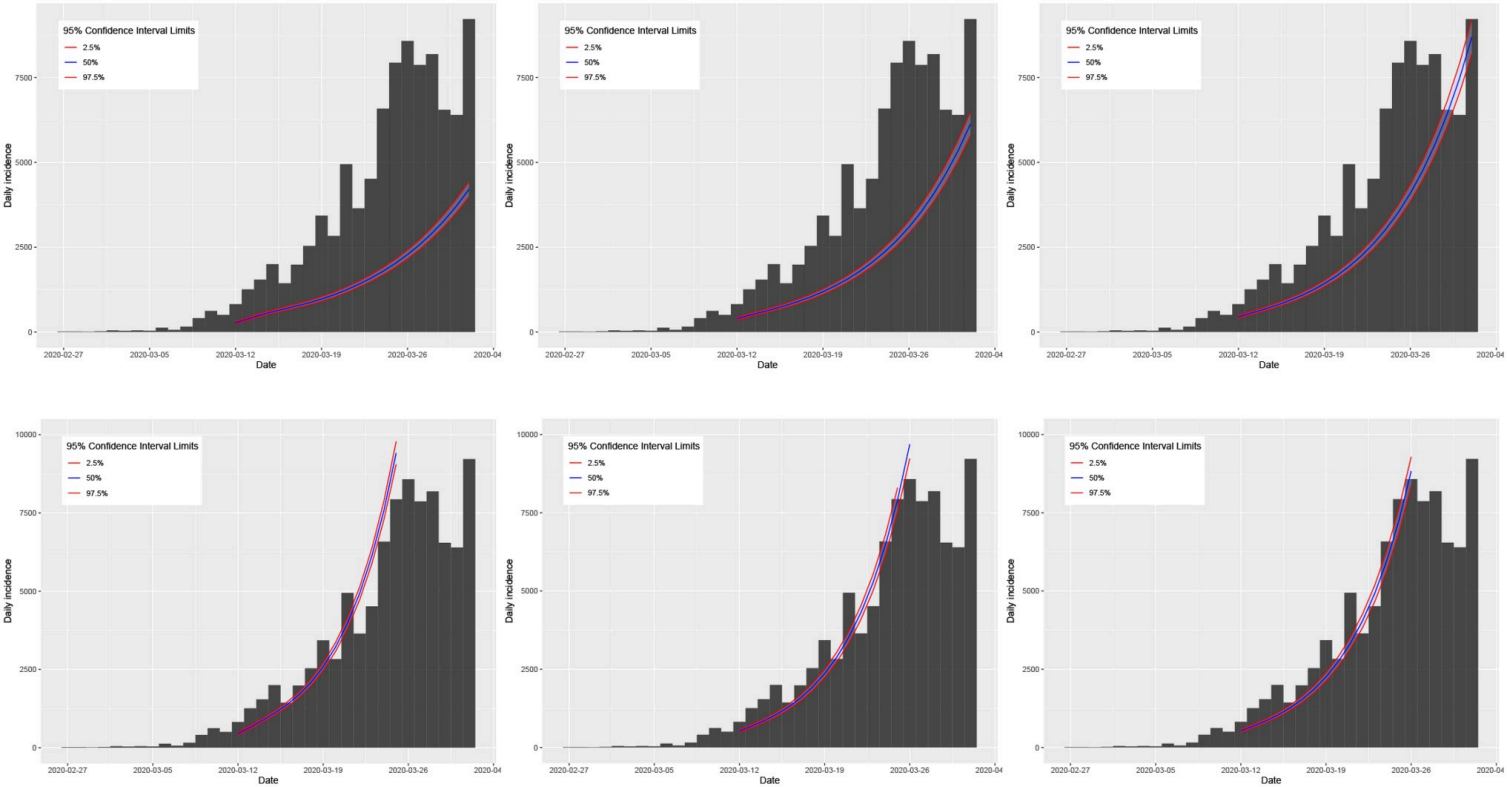
Plots of the observed daily incidence (histograms) in Spain during its growth phase and the predicted daily incidence (purple solid line) estimated using the SIR model (top row) and log-linear model (bottom row) assuming serial interval distributions of *SI*_1_ (left), *SI*_2_ (middle), and *SI*_3_ (right). 95% confidence intervals for the predicted incidence are indicated by the shaded light purple regions.

Plots of the true daily incidence in Italy and Spain during their respective growth phases and the predicted values using the SIR and log-linear models are shown in Figs 19 and 20. In each figure, the first row plots the predictions using the SIR model; the second row plots the predictions using the log-linear model. For the case of Italy, the plots in Fig 19 appear to show that the predictions using the *R*_0_ value estimated from the SIR model and the serial interval of a gamma distribution with mean *μ* = 7.5 and standard deviation *σ* = 3.4 [81] provide the most accurate general predictions. However, although using the *R*_0_ value estimated from the log-linear model generates predictions which are accurate up until the last 7 days of the growth phase (where all three cases show over prediction), these results are more consistent compared with those using the SIR model. For the case of Spain, the plots in Fig 20 show that the predictions using the *R*_0_ value estimated from the SIR model are consistent but significantly under predicting the observed daily incidence. In contrast, predictions using the *R*_0_ value estimated from the log-linear model are consistent and accurate up until the initial peak in daily incidence a few days before the true peak at the end of the growth phase. Based on these results for the growth phase of the outbreak, we propose to use the log-linear model to compute basic predictions for the decay phase.

**Fig 21:**
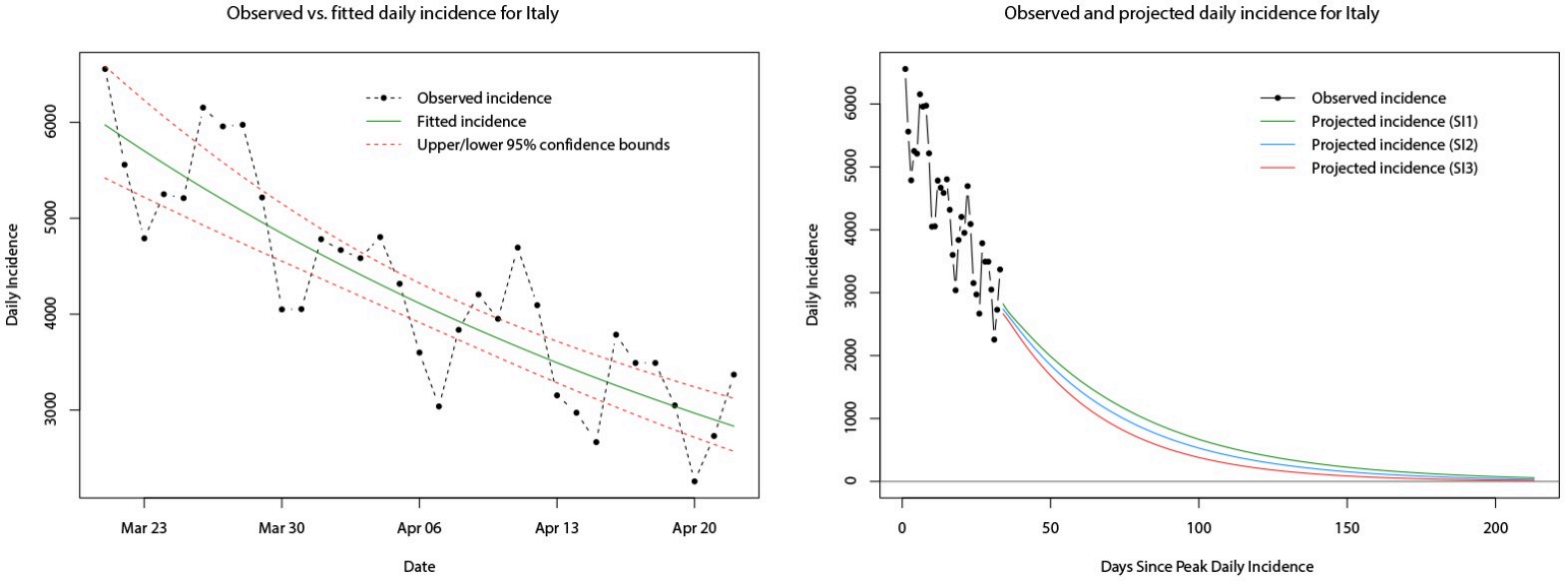
Plots of the observed (dot-dashed black line) and fitted daily incidence (solid green line) for Italy during its decay phase, with upper and lower limits of the 95% confidence interval indicated by the red dashed lines (left). Plots of the observed (dot-dashed black line) and projected daily incidence for the next 180 days using the log-linear model and serial interval distributions *SI*_1_ (green line), *SI*_2_ (blue line), and *SI*_3_ (red line) (right).

**Fig 22:**
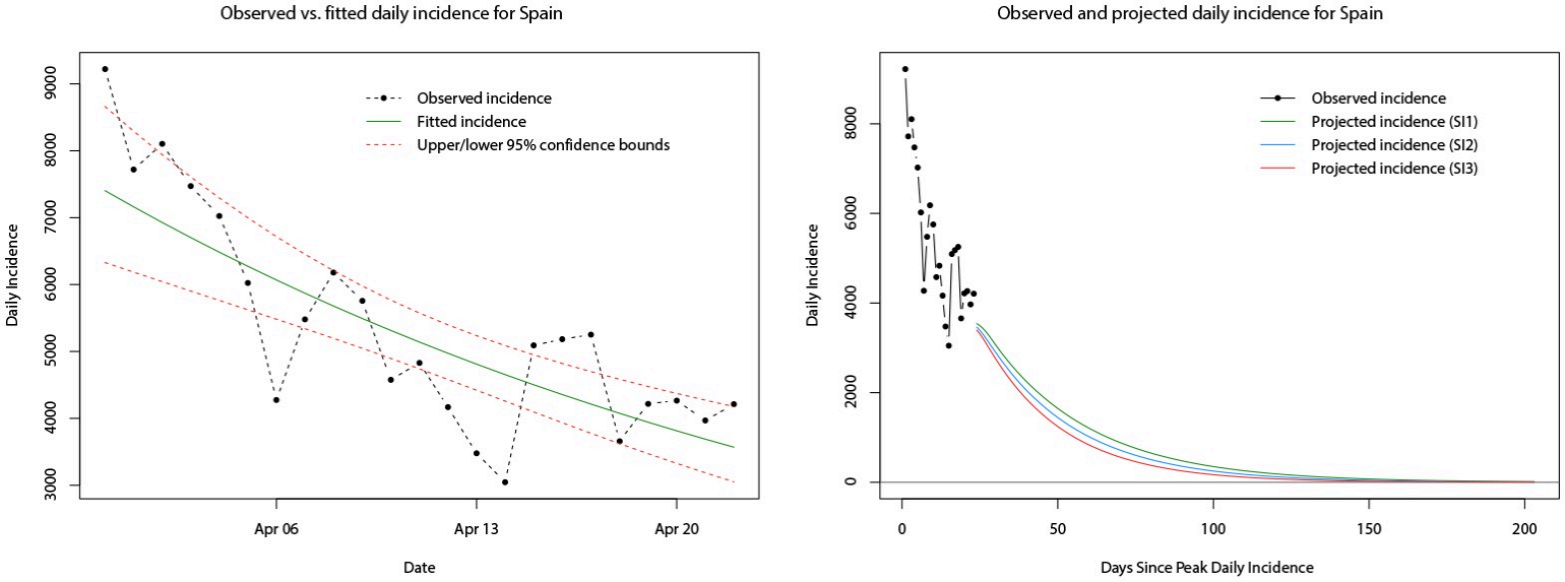
Plots of the observed (dot-dashed black line) and fitted daily incidence (solid green line) for Spain during its decay phase, with upper and lower limits of the 95% confidence interval indicated by the red dashed lines (left). Plots of the observed (dot-dashed black line) and projected daily incidence for the next 180 days using the log-linear model and serial interval distributions *SI*_1_ (green line), *SI*_2_ (blue line), and *SI*_3_ (red line) (right).

At the time of conducting this part of the analysis, approximately one month of daily incidence data was available for the decay phase (following peak daily incidence) of both Italy and Spain. Similarly, we follow the methodology for fitting the log-linear model but now apply it to the decay phase daily incidence. The model is fitted to the decay phase daily incidence in the same way, and model parameters can be computed. Note that for the decay phase, the values and interpretation of the estimated parameters change—the growth rate takes a negative value and the doubling time becomes the halving time (both reflecting the decay and decrease in daily incidence). The fitted log-linear regressions for Italy and Spain are shown in the left hand plots of Figs 21 and 22, respectively. The fitted models appear to provide reasonable fits to the observed decay phase daily incidence much like the case for the growth phase.

Also, as in the growth phase, the *R*_0_ value can still be computed for the log-linear model during the decay phase, and for consistency we obtain mean estimates of *R*_0_ from 10,000 samples of *R*_0_ generated from the log-linear regressions of the daily incidence during the decay phase in conjunction with the three serial distributions. Distributions of these estimates are plotted in S2 Fig and it can be seen that (in contrast to the growth phase) the mean estimates of *R*_0_ for Italy and Spain, individually, are very similar (under the three serial distributions)—between 0.85 and 0.87 for Italy, and 0.77 and 0.83 for Spain. Using the mean estimated *R*_0_ values and the three serial distributions, we computed projections of the daily incidence for the 180 days immediately following the end of the decay phase sample period on 22nd April 2020. The paths of these projections for Italy and Spain are shown in the right hand plots of Figs 21 and 22, respectively.

A simple comparison of the projected daily incidence for both countries is given in Table 6, at one and two months following the end of the decay phase sample period. Observed daily incidence for the remainder of the decay phase was obtained from [44, 90, 91]. In general, it appears that the predictions for future daily incidence (under all three serial distributions) in both Italy and Spain are significantly greater than the observed daily incidence. At the one month time point (21st May 2020) projections of daily incidence for Italy are approximately twice as large as the true incidence; projections of daily incidence for Spain are approximately two to three times as large as the true incidence. Moving forward to the two month time point (21st June 2020) projections of the daily incidence for Italy are approximately two to three times as large as the true incidence; projections of the daily incidence for Spain are up to twice as large as the true incidence. However, the projection of Spanish daily incidence using the serial interval of a gamma distribution with mean *μ* = 6.3 and standard deviation *σ* = 4.2 [86] is almost identical to the true incidence.

**Table 6:** Comparison between the observed and projected daily incidence for Italy and Spain during their respective decay phases, for May and June 2020.

Date	Location	Observed Daily Incidence	Projection (*SI*_1_)	Projection (*SI*_2_)	Projection (*SI*_3_)
21-May-2020	Italy	642	1501	1334	1144
	Spain	593	1502	1301	1105
21-Jun-2020	Italy	224	781	632	468
	Spain	334	593	455	333

Assuming serial interval distributions following a gamma distribution with parameters: i) *μ* = 7.5 and *σ* = 3.4 (*SI*_1_); ii) *μ* = 7 and *σ* = 4.5 (*SI*_2_); iii) *μ* = 6.3 and *σ* = 4.2 (*SI*_3_).

Whilst the results of the projections generally show significant over estimation of future daily incidence in both Italy and Spain, they do provide some additional information to the reproduction values regarding the trends of daily incidence. However, such forecasts should be not be taken directly at face value as there are a number of pitfalls that will influence the predictions. Limited decay phase incidence data was available at the time of the original analysis, which is likely to have led to less accurate estimates of *R*_0_ and thus predictions. On a related note, the predictions are conditional on the data up until the end of the sample decay phase data and thus do not account for any health policies or interventions implemented after this, likely leading to the over estimation.

## Conclusion

In this paper, we have provided a simple statistical analysis of the novel Coronavirus (COVID-19) outbreak in Italy and Spain—two of the worst affected countries in Europe. Using data of the daily and cumulative incidence in both countries over approximately the first month after the first cases were confirmed in each respective country, we have analysed the trends and modelled the incidence and estimated the basic reproduction value using two common approaches in epidemiology—the SIR model and a log-linear model.

Results from the SIR model showed an adequate fit to the cumulative incidence of Spain and its most affected regions in the early stages of the outbreak, however, it showed significant under estimation in the case of Italy and its most affected regions. Estimates of the basic reproduction number in the early stage of the outbreak from the model were found to be greater than one in all cases, suggesting a growing infectiousness of COVID-19—in line with expectations. Applying the log-linear regression model to the daily incidence, results for the growth phase of the outbreak in Italy and Spain revealed a greater growth rate for Spain compared with Italy (and their most affected regions)—approximately between 0.21 to 0.24 for the former and 0.15 to 0.18 for the latter. The time for the daily incidence to double for Spain was also found to be shorter than Italy (approximately three days compared to four days).

With the lack of detailed clinical COVID-19 data for the two countries, we utilised existing results regarding the serial interval distribution of COVID-19 from the literature to estimate the basic reproduction number via the log-linear model. Estimates of this value were found to be between 2.1 and 3 for Italy and its most affected region Lombardy, and between 2.5 and approximately 4 for Spain and its most affected regions of Madrid and Catalonia. Further analysis of the effective reproduction number (based on the incidence over the previous seven days) indicated that in both countries the infectious of COVID-19 was decreasing and reflecting the positive impact of health interventions such as nationwide lock downs.

Basic predictions of future daily incidence in Italy and Spain were estimated using the log-linear regression model for the decay phase of the outbreak. Estimates of the projected daily incidence at various time points in the future were generally found to be between two to three times larger than the true levels of daily incidence. These results highlight the fact that the estimates may only give reasonable indications in the short term, since they are based on past data which may or may not account for factors which change in the short term—e.g. new health interventions, public policy, etc.

Despite the simplicity of our results, we believe that they provide an interesting insight into the statistics of the COVID-19 outbreak in two of the worst affected countries in Europe. Our results appear to indicate that the log-linear model may be more suitable in modelling the incidence of COVID-19 and other infectious diseases in both the growth and decay phases, and for short term predictions of the growth (or decay) of the number of new cases when no intervention measures have recently been implemented. In addition, the results could be useful in contributing to health policy decisions or government interventions—especially in the case of a significant second wave of COVID-19. However, these results should be used in conjunction with the results from other more complex mathematical and epidemiological models.

## Supporting information

S1 Fig Plots of the distributions of samples of *R*_0_ values computed from the fitted log-linear regressions of growth phase incidence. i) Lombardy (top left); ii) Italy (top right); iii) Madrid (middle left); iv) Catalonia (middle right); v) Spain (bottom). a) *SI*_1_ (blue); b) *SI*_2_ (red) c) *SI*_3_ (green).
(TIF)

S2 Fig Plots of the distributions of samples of *R*_0_ values computed from the fitted log-linear regressions of decay phase incidence. i) Italy (left); ii) Spain (right). a) *SI*_1_ (green); b) *SI*_2_ (red) c) *SI*_3_ (blue).
(TIF)
