# Supplementary figures and images for: A statistical analysis of the novel coronavirus (COVID-19) in Italy and Spain
Source: PLoS One. 2021 Mar 25;16(3):e0249037. doi: 10.1371/journal.pone.0249037 (PMC7993852; doi:10.1371/journal.pone.0249037)

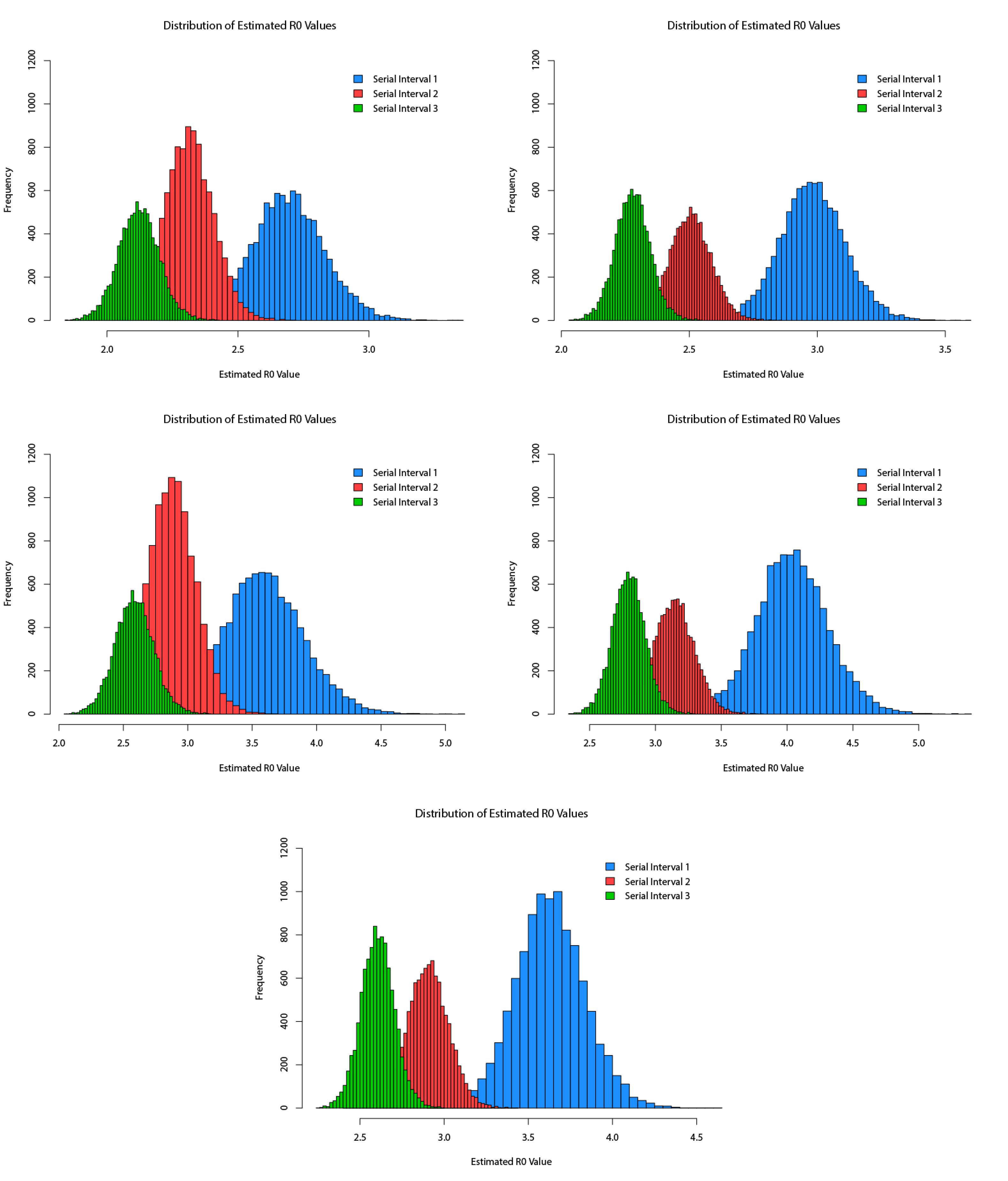

Supplement: S1 Fig — i) Lombardy (top left); ii) Italy (top right); iii) Madrid (middle left); iv) Catalonia (middle right); v) Spain (bottom). a) SI1 (blue); b) SI2 (red) c) SI3 (green). (TIF) [file pone.0249037.s001.tif]

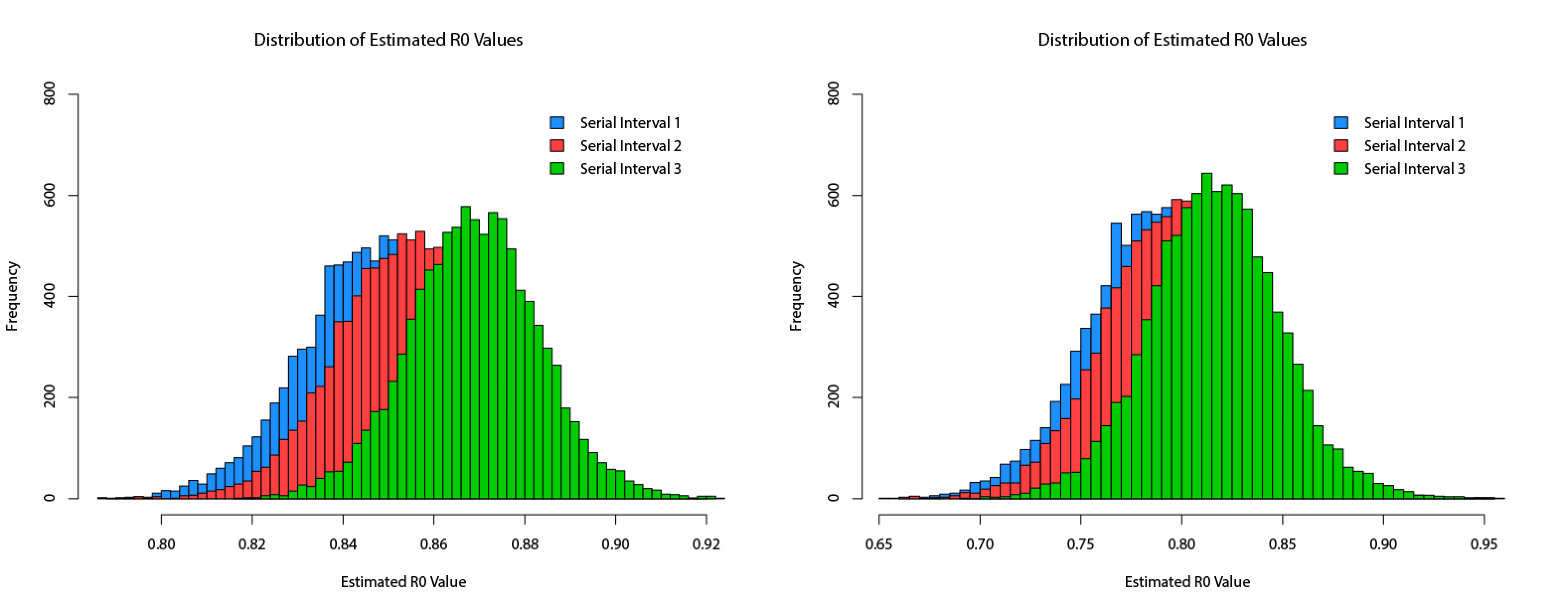

Supplement: S2 Fig — i) Italy (left); ii) Spain (right). a) SI1 (green); b) SI2 (red) c) SI3 (blue). (TIF) [file pone.0249037.s002.tif]
